# Activation of heme oxygenase-1 by Ginkgo biloba extract differentially modulates endothelial and smooth muscle-like progenitor cells for vascular repair

**DOI:** 10.1038/s41598-019-53818-7

**Published:** 2019-11-21

**Authors:** Tao-Cheng Wu, Jia-Shiong Chen, Chao-Hung Wang, Po-Hsun Huang, Feng-Yen Lin, Liang-Yu Lin, Shing-Jong Lin, Jaw-Wen Chen

**Affiliations:** 1 Division of Cardiology, Department of Medicine, Taipei Veterans General Hospital, Taipei, Taiwan; 2 Cardiovascular Research Center, National Yang-Ming University, Taipei, Taiwan; 3 Division of Cardiology, Department of Internal Medicine, Chang Gung Memorial Hospital, Keelung, Taiwan; 4 Institute of Clinical Medicine, National Yang-Ming University, Taipei, Taiwan; 5 Department of Internal Medicine, School of Medicine, College of Medicine, Taipei Medical University, Taipei, Taiwan; 6 Division of Endocrinology and Metabolism, Department of Medicine, Taipei Veterans General Hospital, Taipei, Taiwan; 7 Department of Medical Research, Taipei Veterans General Hospital, Taipei, Taiwan; 8 Precision Medicine Research Center, Taipei Veterans General Hospital, Taipei, Taiwan; 9 Institute of Pharmacology, National Yang-Ming University, Taipei, Taiwan

**Keywords:** Clinical pharmacology, Drug development

## Abstract

Vascular progenitors such as endothelial progenitor cells (EPCs) and smooth muscle-like progenitor cells (SMPCs) may play different roles in vascular repair. *Ginkgo biloba* extract (GBE) is an exogenous activator of heme oxygenase (HO)-1, which has been suggested to improve vascular repair; however, the detailed mechanisms have yet to be elucidated. This study aimed to investigate whether GBE can modulate different vascular progenitor cells by activating HO-1 for vascular repair. A bone marrow transplantation mouse model was used to evaluate the *in vivo* effects of GBE treatment on wire-injury induced neointimal hyperplasia, which is representative of impaired vascular repair. On day 14 of GBE treatment, the mice were subjected to wire injury of the femoral artery to identify vascular reendothelialization. Compared to the mice without treatment, neointimal hyperplasia was reduced in the mice that received GBE treatment for 28 days in a dose-dependent manner. Furthermore, GBE treatment increased bone marrow-derived EPCs, accelerated endothelial recovery, and reduced the number of SMPCs attached to vascular injury sites. The effects of GBE treatment on neointimal hyperplasia could be abolished by co-treatment with zinc protoporphyrin IX, an HO-1 inhibitor, suggesting the *in vivo* role of HO-1. In this *in vitro* study, treatment with GBE activated human early and late EPCs and suppressed SMPC migration. These effects were abolished by HO-1 siRNA and an HO-1 inhibitor. Furthermore, GBE induced the expression of HO-1 by activating PI3K/Akt/eNOS signaling in human late EPCs and via p38 pathways in SMPCs, suggesting that GBE can induce HO-1 *in vitro* through different molecular mechanisms in different vascular progenitor cells. Accordingly, GBE could activate early and late EPCs, suppress the migration of SMPCs, and improve *in vivo* vascular repair after mechanical injury by activating HO-1, suggesting the potential role of pharmacological HO-1 activators, such as GBE, for vascular protection in atherosclerotic diseases.

## Introduction

Accumulating evidence suggests that circulating progenitor cells contribute to vascular healing and remodeling in response to vascular injury^1–4^. When the vascular endothelium is injured by mechanical stress, bone marrow-derived vascular progenitor cells are recruited to the site of luminal injury. Under physiological conditions, vascular progenitor cells differentiate into endothelial cells, contributing to reendothelialization and accelerating vascular healing^5,6^. However, the participation of vascular progenitor cells in vascular repair is impaired by factors such as advanced age, diabetes mellitus, hypertension, and smoking. In such conditions, vascular progenitor cells can also differentiate into vascular smooth muscle cells (VSMCs) or macrophages, thereby promoting the formation of atherosclerotic lesions instead of vascular repair at the site of injury^6,7^.

Adult peripheral blood and bone marrow contain sub-populations of vascular progenitor cells that can be differentiated into early (out-growth) endothelial progenitor cells (EPCs) and smooth muscle-like progenitor cells (SMPCs) by culturing in a supplemented microvascular endothelial growth medium^8–10^. Previous studies have reported that late EPCs exhibit better proliferation, migration, and tube formation function than early EPCs^11^. In addition, early EPCs have been shown to act as cytokine donors, and a hindlimb ischemia model of mechanical injury and vascular regeneration showed that late EPCs could replace damaged endothelial cells^12–14^. Although their properties have not been completely elucidated and further definitions of the cell markers are still required, SMPCs are known to express smooth muscle contraction protein α-SMA with a morphology similar to that of VSMCs. They have also been shown to play a role both in the acceleration of atherosclerosis following vascular injury and in vascular regeneration in hindlimb ischemia^7,12,15,16^.

Several growth factors, including vascular endothelial growth factor (VEGF) and stromal cell-derived factor-1, are secreted into the circulation at the sites of vascular injury. These growth factors serve as chemoattractants for the recruitment of bone marrow-derived vascular progenitor cells^17–19^. The contributions of stromal cell-derived factor-1 and VEGF to angiogenesis and vascular repair have been reported to require heme oxygenase-1 (HO-1), a 32-kD stress-inducible enzyme^20,21^. HO-1 metabolizes heme to generate carbon monoxide, biliverdin and iron, all of which have anti-inflammatory and antioxidant effects. HO-1 induction occurs as an adaptive response to tissue injury, and it has been shown to promote EPC migration, wound healing, and angiogenesis mediated through one or more heme degradation products^20,22^. However, the detailed mechanisms by which HO-1 modulates different circulating vascular progenitor cells such as late EPCs and SMPCs for vascular repair have not been clarified.

*Ginkgo biloba* extract (GBE) 761 is a standardized extract of Ginkgo biloba leaves which has been shown to exhibit a wide variety of biological activities, including anti-inflammatory and antioxidant effects^23,24^. We previously showed that GBE can inhibit the proliferation of cultured VSMCs and decrease intimal responses to balloon injuries of the abdominal aorta in cholesterol-fed rabbits^25^. GBE has also been shown to improve vascular repair *in vivo* and to activate EPCs *in vitro*^26,27^. Recently, we further demonstrated the induction of HO-1 by GBE in mice peripheral mononuclear cells and human aortic endothelial cells^28^. However, the role of GBE-mediated upregulation of HO-1 in vascular progenitor cells is not known. Therefore, we conducted this study to test the hypothesis that HO-1 induced by GBE may modulate different vascular progenitor cells for vascular repair.

## Methods

### Animal experiments

All mice were purchased from the Jackson Laboratory (Bar Harbor, ME, USA), and kept in microisolator cages on a 12-h day/night cycle. All experimental procedures and protocols were conducted in accordance with and approved by the Institutional Animal Care Committee of National Yang-Ming University, Taipei, Taiwan (IACCU number: 2015-126), and complied with the Guide for the Care and Use of Laboratory Animals.

### Mouse femoral artery wire-injury model

A mouse femoral artery wire-injury model was used to evaluate the contribution of EPCs to neointimal formation. The wire-injury model was performed as previously described^29^. Transluminal injury of the femoral artery was performed under a dissecting microscope. In brief, either the left or right femoral artery was exposed by blunt dissection. The accompanying femoral nerve was carefully separated, and the femoral vein was isolated from the artery^1^. The femoral artery and vein were then looped together proximally and distally with 6-0 silk sutures to allow for temporary vascular control during the procedure. A small branch between the rectus femoris and vastus medialis muscles was isolated, and looped proximally and ligated distally with 6-0 silk sutures. The vein and connective tissues around the artery were carefully removed with microsurgical forceps. The exposed muscular branch artery was dilated by the topical application of one drop of 1% lidocaine hydrochloride, and a transverse arteriotomy was performed on this branch. A straight spring wire (0.38 mm in diameter, no. C-SF-15-15, Cook, Bloomington, IN, USA) was carefully inserted into the femoral artery toward the iliac artery. The wire was left in place for 1 minute to denude and dilate the artery. The wire was then removed, and a silk suture looped at the proximal portion of the muscular branch artery was secured. Blood flow to the femoral artery was restored by releasing the sutures placed in the proximal and distal femoral portions. The skin incision was closed with 5-0 silk sutures. At different time points, the femoral arteries were excised, fixed in OCT compound (TissueTek, Tokyo, Japan), and used for immunofluorescence studies.

### Mobilization of EPCs in GBE-treated wild-type mice

To examine the effect of EPC mobilization in response to stimulation with GBE (100 mg·kg^−1^·day^−1^) and ZnppIX (HO-1 inhibitor, 2.5 mg·kg-1·day-1), the fluorescein isothiocyanate anti-mouse Sca-1 (eBioscience, San Diego, CA, USA) and phycoerythrin anti-mouse Flk-1 (vascular endothelial growth factor receptor-2, eBioscience) antibodies were used. The number of Sca-1^+^/Flk-1^+^ cells in peripheral blood mononuclear cells (PBMNCs) was examined using a fluorescence-activated cell sorter (FACS Calibur; Becton Dickinson, San Jose, CA, USA). Circulating EPCs were quantified by counting the Sca-1+/Flk-1+ cells.

### Bone marrow transplantation model

Bone marrow transplantation was done as previously described^29^. In brief, recipient wild-type mice at 8 weeks of age were lethally irradiated with a total dose of 9.0 Gy. Enhanced green fluorescent protein (eGFP) transgenic mice (C57B/6 J background) that ubiquitously expressed eGFP (Level Biotechnology Inc., Taipei, Taiwan) 21 were used. After being irradiated, the recipient mice received unfractionated bone marrow cells (5 × 10^6^) from the eGFP mice by tail vein injection. Eight weeks after the bone marrow transplantation, the chimeric mice were subjected to wire injury (n = 6 per group). Repopulation with eGFP-positive bone marrow cells was measured using flow cytometry to be 95%. Twelve days after the induction of wire injury in the bone marrow-reconstituted mice, tissues were harvested for confocal microscope (Leica TCS SP2 AOBS, Mannheim, Germany) and histological analyses. Vascular reendothelialization was evaluated in the frozen sections (5 μm) of the gastrocnemius muscle from the limbs in which the wire injury had been performed. Bone marrow-derived EPCs were stained with antibodies directed against eGFP (Chemicon, Temecula, CA, USA), α-SMA (Sigma, St. Louis, MO, USA), and von Willebrand factor (vWF) (DAKO, produced in rabbits, Sydney, Australia). The density of bone marrow-derived EPCs was assessed by counting eGFP+/vWF+ double-positive cells (cyan color), and the density of SMPCs was estimated by counting eGFP+/α-SMA+ double-positive cells (yellow color) under high power fields (x100) in at least six different cross-sections from different animals. Nuclei were stained with DAPI or Hoechst 33258 (Sigma, St. Louis, MO, USA).

### EPC and SMPC isolation, cultivation, and characterization

EPCs and SMPCs were cultured and identified according to the protocol described in a previous study^30,31^. Total mononuclear cells (MNCs) were isolated from 30 mL of peripheral blood from human volunteers (the Human Investigation Committee of Cheng-Hsin Rehabilitation Medical Center approved the study protocol (CHGH-IRB:(136) 97-17-2) by density gradient centrifugation with Histopaque-1077 (density 1.077 g/mL, Sigma-Aldrich, St. Louis, MO, USA). MNCs (5 × 10^6^) were placed on fibronectin-coated 6-well plates (Chemicon, Temecula, CA, USA) in 2 mL of endothelial growth medium (EGM-2 MV, Cambrex, Walkersville, MD, USA) supplemented with hydrocortisone, R3-insulin-like growth factor 1, human endothelial growth factor, VEGF, human fibroblast growth factor, gentamicin, amphotericin B, vitamin C, and 20% fetal bovine serum (Supplement Fig. 1A), and cultured at 37 °C in a 5% CO2 incubator. After 4 days of culture, the media was changed and non-adhered cells were removed. Upon observation, the attached early EPCs were elongated with a spindle shape (Supplement Fig. 1B). The media was replaced every 4 days and each colony/cluster was monitored. After 2 to 4 weeks of MNC culture, a certain number of vascular progenitor cells continued to grow into colonies of late EPCs or SMPCs. The late EPCs exhibited a “cobblestone” morphology and a monolayer growth pattern typical of mature endothelial cells at confluence (Supplement Fig. 1C,D)^11^. The SMPCs had a spindle-like shape and were similar to mature SMCs at confluence. Cells under passage 3 were used for cell functional studies (Supplement Fig. 1E,F).

The early EPCs were characterized as adherent cells, which were positive for both acetylated low-density lipoprotein (ac-LDL) uptake and lectin binding in direct fluorescent staining (Supplement Fig. 1G)^32^. In brief, adherent cells were first incubated with 2.4 μg/mL 1,1′-dioctadecyl-3,3,3′,3′-tetramethylindocarbocyanine-labeled ac-LDL (DiI-ac-LDL; Molecular Probes Inc., Eugene, OR, USA) for 1 hour, and fixed in 4% paraformaldehyde. The late EPC-derived outgrowth endothelial cells and SMPCs were characterized by immunofluorescence staining for the expression of PECAM-1 (CD31) (Santa Cruz Biotechnology, Santa Cruz, CA, USA), and α-SMA (Sigma-Aldrich, St. Louis, MO, USA) (Supplement Fig. 1H,I). The fluorescent images were recorded using a laser scanning confocal microscope. Monocytes, early EPCs, late EPCs, and SMPCs were also analyzed for the expression of cell-surface antigens using labeling with Fluorescein fluorescent dye-, R-phycoerythrin-, peridinin chlorophyll protein complex-, and allophycocyanin-conjugated mAbs, and direct four-color flow cytometry analysis (FACScan, Becton Dickinson, Sunnyvale, CA, USA), as previously described^33,34^. Briefly, 1 × 10^5^ late EPCs and SMPCs were incubated with 10 μL of fluorescein isothiocyanate-conjugated anti-human CD34 mAb (Biolegend, San Diego, CA, USA), 3 μL of R-phycoerythrin-conjugated anti-human mAb CD133 (Miltenyi Biotec Ltd., Surrey, UK), 10 μL of peridinin chlorophyll protein complex-conjugated anti-human CD45 mAb (Becton Dickinson, Sunnyvale, CA, USA), and 10 μL of allophycocyanin-conjugated anti-human KDR mAb (R&D Systems Inc., Minneapolis, MN, USA) at 4 °C for 30 minutes prior to FACS analysis (data not shown). Control isotype immunoglobulin G1 and G2a antibodies were obtained from Becton Dickinson.

### EPC differentiation and proliferation assay

After seeding the PBMNCs, cells were incubated with a fixed concentration of GBE for 2 or 4 days. Quantification of early EPCs was performed by counting the cells that were positive for both lectin-binding and Dil-ac-LDL staining in six random high-power (x100) microscope fields.

The proliferation of early EPCs, late EPCs, and SMPC was determined using the 3-(4,5-dimethylthiazol-2-yl)-2,5-diphenyltetrazolium bromide (MTT) assay. Cells were cultured with various concentrations of GBE or hemin (an HO-1 activator, Sigma-Aldrich, St. Louis, MO, USA). After the indicated culture times, MTT (0.5 mg/mL, Sigma-Aldrich, St. Louis, MO, USA) was added to each well, and the cells were incubated for an additional 4 hours. The blue formazan crystals were dissolved with dimethyl sulfoxide and absorbance was measured at 570/650 nm for quantification.

### EPC fibronectin adhesion assay

Early EPCs were incubated with increasing concentrations of GBE for 24 hours. Early EPCs were then detached with trypsin/ethylenediaminetetraacetic acid (EDTA) and 1 × 10^4^ cells were placed in fibronectin-coated 24-well plates for 30 minutes at 37 °C. Early EPCs were quantified by counting the adherent cells in six random high-power (x100) microscope fields.

### Cell migration assay

The migratory ability of both late EPCs and SMPCs in response to VEGF was assessed using a modified Boyden chamber assay (Transwell; Coster, Cambridge, MA, USA)^35^. In brief, isolated early EPCs, late EPCs, and SMPCs were detached with trypsin/EDTA, and then 1 × 10^4^ cells with VEGF-free EGM-2 MV were placed in the upper chamber of 24-well Transwell plates with polycarbonate membranes (8-μm pores). EGM-2 MV medium supplemented with VEGF (50 ng/mL) was placed in the lower chamber. After incubation for 24 hours, the membranes were washed briefly with phosphate-buffered saline and fixed in 4% paraformaldehyde. The upper part of each membrane was wiped gently with a cotton ball and the membrane was stained using a hematoxylin solution. The number of cells that had migrated were counted in six random high-power (x100) microscope fields.

### *In vitro* wound healing migration assay

A wound healing migration assay was performed as previously reported with minor modifications^36^. SMPCs with a number of 1 × 10^5^ cells were seeded onto 6-well plates with maintenance medium until they reached confluency after 24 h. Scratch wounds ~1 mm wide were created by 1000 μl tip. (After gentle washing of the detached cells with PBS, the growth medium was changed to fresh medium. The pictures of wound closure were taken at 6 and 12 h during post-scratching at 100× magnification under a microscope (Olympus, Tokyo, Japan). The cell migration was calculated using the ImageJ software program (NIH, MD, USA).

### EPC tube formation assay

The tube formation assays of late EPCs were assessed using an *In Vitro* Angiogenesis Assay Kit (Chemicon, Temecula, CA, USA) according to the manufacturer’s instructions. Briefly, ECMatrix gel solution was thawed at 4 °C overnight, and then mixed with ECMatrix diluent buffer and placed in a 96-well plate at 37 °C for 1 hour to allow the matrix solution to solidify. Late EPCs were treated with 100 μg/mL GBE for 24 hours and harvested with trypsin/EDTA. The EPCs (1 × 10^4^ per well) were then placed on the matrix solution along with 100 μL EGM-2 MV medium and incubated at 37 °C for 16 hours. For inhibitor studies, the cells were incubated with or without GBE, detached, and plated on Matrigel (Chemicon, Temecula, CA, USA) with ZnPPIX (1 μM to 5 μM) or LY294002 (10 μM) at 37 °C for 16 hours. After incubation, tubule formation was evaluated under an inverted light microscope (x100) by counting the junction points in random high-power (x100) microscope fields from four independent experiments.

### Western blot analysis

Early EPCs, late EPCs, and SMPCs were lysed in lysis buffer (62.5 mM Tris-HCl, 2% sodium dodecyl sulfate, 10% glycerol, 0.5 mM phenylmethanesulfonyl fluoride (PMSF), 2 μg/mL aprotinin, pepstatin, and leupeptin), as previously described^32^. Proteins in the cell lysates were separated using sodium dodecyl sulfate-polyacrylamide (10%) gel electrophoresis, followed by transfer onto poly(vinylidene fluoride) membranes. The membranes were probed with monoclonal antibodies against phosphorylated endothelial nitric oxide synthase (eNOS) (Upstate Biotechnology, Lake Placid, NY, USA), eNOS (Upstate Biotechnology), HO-1 (Affinity BioReagents Inc., Golden, CO, USA), β-actin (Chemicon, Temecula, CA, USA), phosphorylated protein kinase B (Akt), and Akt (Cell Signaling Technology, Beverly, MA, USA). Bound antibodies were visualized using chemiluminescence detection reagents. Protein band densitometry was measured using ImageQuant software (Promega, Madison, WI, USA).

### Measurement of reactive oxygen species (ROS) production

ROS production in EPCs was determined using a fluorometric assay with 2′,7′-dichlorofluorescin diacetate (DCFH-DA) as a probe to detect the presence of H2O2. The fluorescence intensity was measured at an excitation wavelength of 485 nm and emission wavelength of 530 nm using a fluorescent microplate reader (VICTPR2 Multilabel Readers, USA).

### Measurement of nitric oxide (NO) production

NO production in EPCs was determined using a fluorometric assay with 4-amino-5-methylamino-2′,7′-difluorescein (DAF-FM) as a probe to detect the presence of NO. The fluorescence intensity was measured at an excitation wavelength of 485 nm and emission wavelength of 530 nm using a fluorescent microplate reader (VICTPR2 Multilabel Readers, USA).

### Gene silencing using small inhibitory RNAs (siRNAs)

HO-1 and eNOS small inhibitory RNAs (siRNAs) were purchased from Santa Cruz (Santa Cruz Biotechnology, Santa Cruz, CA, USA). Cells were incubated with 25 or 50 nM siRNA. Scrambled siRNA (Dharmacon, Lafayette, CO, USA) was used as a control. The silencing protocol used multiple transfection procedures. Specific siRNAs were incubated with 8 μL Oligofectamine solution (Invitrogen, Carlsbad, CA, USA) and added to the antibiotic and serum-free medium for 6 hours, followed by pulsing with normal medium, incubation for 2 days, and repeated one time. After incubation with GBE for 24 hours, the HO-1 expression level and cell migration were analyzed. The efficiency of specific HO-1 and eNOS siRNA inhibition was verified by Western blot using lysates from cells that were harvested on day 4.

### Statistical analysis

All data were expressed as mean ± standard error of the mean (SEM) for continuous variables and as the number (%) for categorical variables. Statistical analysis was performed using an unpaired Student’s t test or one-way ANOVA. A P value of < 0.05 was considered to be statistically significant. All statistical analyses was using SPSS software (version 12, SPSS, Chicago, IL, USA).

### Ethics approval and consent to participate

Institutional Animal Care Committee of National Yang-Ming University, Taipei, Taiwan (IACCU number: 2015–126), and complied with the Guide for the Care and Use of Laboratory Animals.

## Results

### Wire injury-induced neointimal hyperplasia was reduced by GBE

**Figure 1:**
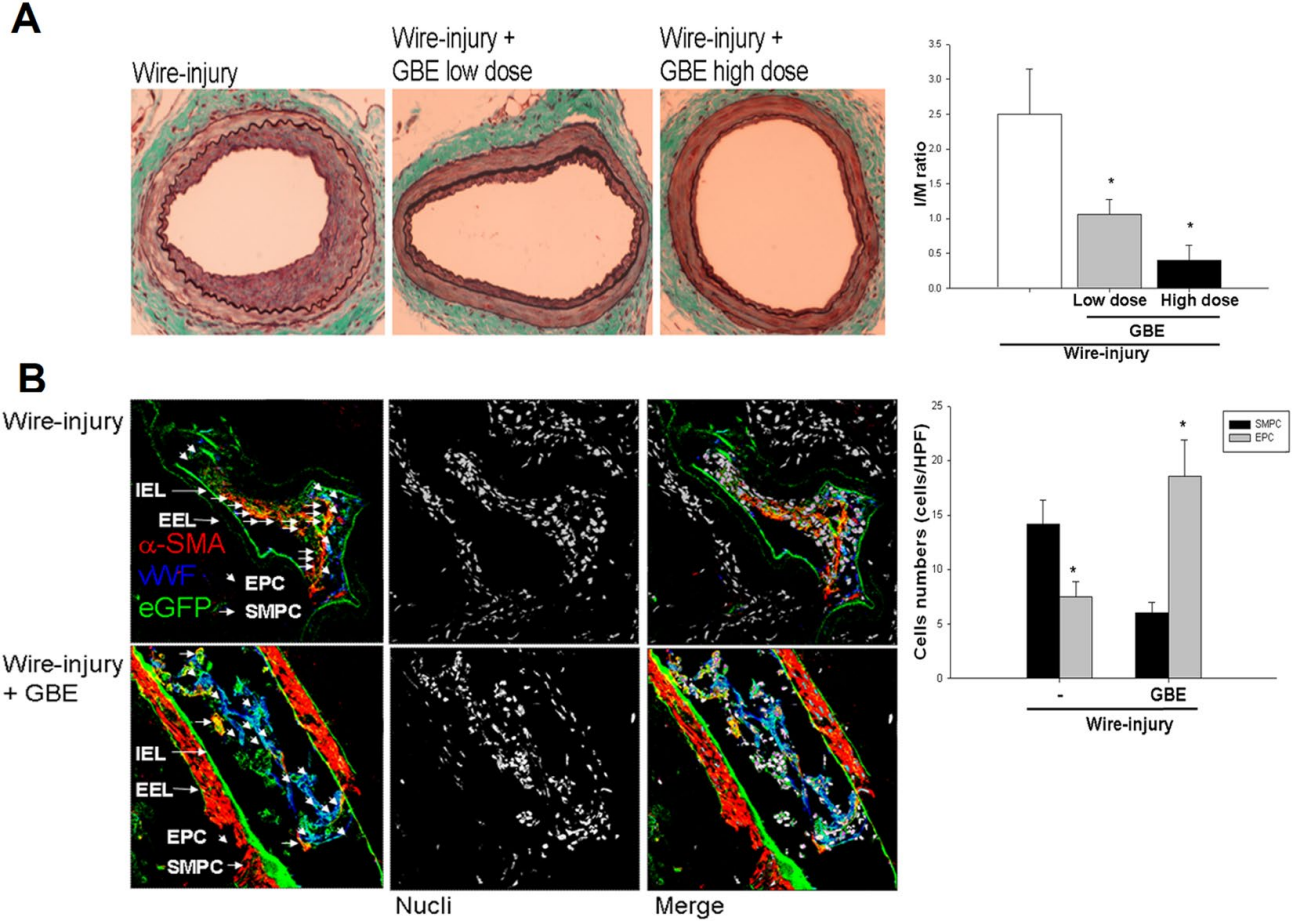
Effects of GBE on the accumulation of bone marrow-derived vascular progenitor cells on injured vessel walls and vascular intima/media ratio after wire injury. (**A**) Femoral arteries were harvested from C57BL/6 mice fed with water, low-dose GBE (100 mg·kg-1·day-1), or high-dose GBE (300 mg·kg-1·day-1). Masson trichrome staining was performed on mouse femoral arteries harvested 28 days after wire injury (n = 6 each in three groups, ×400). Quantification of the intima/media ratio showed the dose-dependent effects of GBE treatment in the mice after wire injury. (**B**) Femoral arteries were harvested from C57BL/6 mice treated with or without GBE 12 days after wire injury. Confocal immunofluorescent images (red for a-SMA, blue for vWF, green for eGFP, and white for cell nuclei) showed the accumulation of different bone marrow-derived vascular progenitor cells, including EPCs (cyan, short arrowheads) and SMPCs (yellow, arrows) on the walls of injured vessels. Quantification of the number of EPCs (cyan) and SMPCs (yellow) showed that the accumulation of SMPCs on the injured vessel walls was significantly reduced, while that of EPCs was increased in the presence of GBE compared with the controls. EEL: external elastic lamina; L: lumen; I: intima; IEL: internal elastic lamina; M: media. Data are expressed as mean ± SEM; n = 6. Scale bars represent 50 μm. *P < 0.05 compared with the control group; ^#^P < 0.05 vs. the GBE-treated group. (n = 6 in each group).

To investigate the contribution of EPCs to neointimal hyperplasia, a mouse femoral artery wire-injury model was used. Before the vascular injury was performed, the mice were given water containing GBE (100 or 300 mg·kg^−1^·day^−1^) for 4 weeks. On day 28 after the wire injury of the femoral artery, the mice were sacrificed, and the neointimal growth of the injured vessels was evaluated by histological analysis (Fig. 1A). Intimal hyperplasia, as indicated by the intima/media ratio, was reduced in the mice that received GBE in a dose-dependent manner. These results suggested that GBE treatment prevented wire injury-induced neointima formation and promoted vascular repair. Wire injury-stimulated neointimal hyperplasia was reduced by GBE.

### GBE enhanced the attachment of bone marrow-derived EPCs but suppressed that of bone marrow-derived SMPCs to the walls of injured vessels

To clarify whether the activation of EPCs by GBE plays a crucial role in vascular repair, a bone marrow transplantation approach was used. After pretreatment with GBE for 2 weeks, recipient mice received bone marrow from GFP transgenic mice, and were then subjected to wire injury of the femoral artery. On day 12 after the wire injury, cells with remarkable double expressions of both GFP + and vWF (an indicator of endothelial cells) were defined as bone marrow-derived EPCs (or endothelial cells), and cells with both GFP + and α-SMA + (indicating VSMCs) were defined as bone marrow-derived SMPCs (or VSMCs), which adhered to the surfaces of the injured vessel walls (Fig. 1B). The numbers of adhered EPCs and SMPCs were then quantified in the mice treated with or without GBE. On day 12 after vascular injury, in the mice without GBE treatment, 1.8 times more SMPCs were found to have accumulated on the surfaces of the injured vascular walls than EPCs (Fig. 1B; P < 0.05). In contrast, in the mice with GBE treatment, 3.6 times more EPCs were found to have accumulated on the surfaces of the injured vascular walls than SMPCs (P < 0.05). Furthermore, the neointima/media ratio was increased after wire injury (P < 0.05), but was reduced in the mice with GBE treatment compared to those without GBE treatment (Fig. 1A; P < 0.05). Therefore, GBE treatment prevented the homing and accumulation of bone marrow-derived SMPCs and promoted bone marrow-derived EPCs on the injured vascular walls.

### The enhanced effects of GBE on mobilization of EPC-like cells into the circulation in response to wire injury were abolished by ZnPPIX

**Figure 2:**
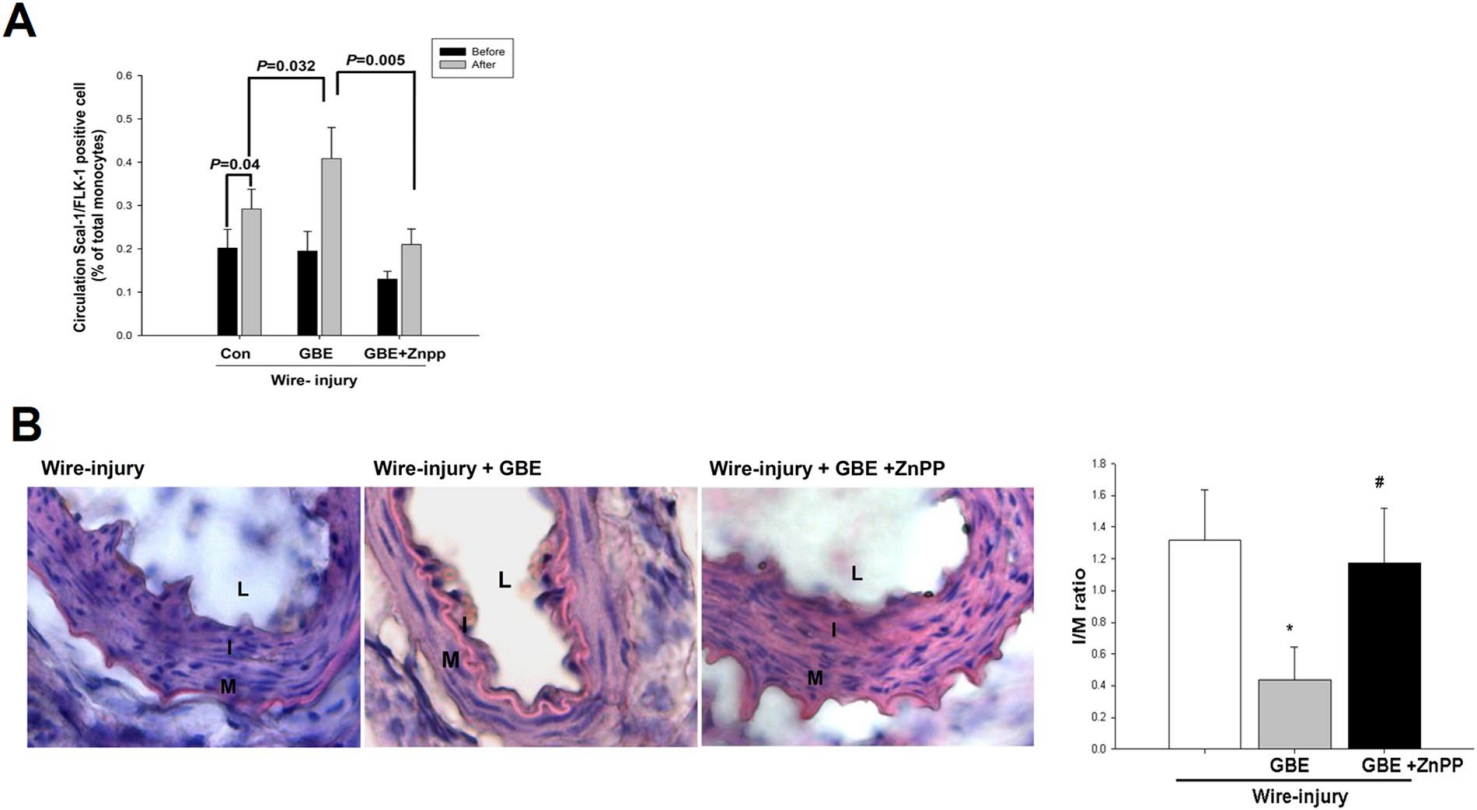
Effects of GBE on mobilization of EPC-like cells into the circulation and vascular neointimal formation in response to wire injury were abolished by ZnPPIX. (**A**) Quantitative analysis of the number of circulating EPC-like cells (Sca-1+/Flk-1+ cells) was performed in the peripheral blood of C57BL/6 mice before and 4 days after wire injury of the femoral arteries. Circulating EPCs were increased after wire injury in the mice treated with water, GBE, or GBE combined with ZnppIX. Compared to the mice treated with water or with both GBE and ZnppIX, circulating EPCs were significantly increased in the mice with GBE treatment after wire injury. (**B**) Femoral arteries were harvested from C57BL/6 mice fed with water, GBE, or GBE combined with ZnPPIX. Hematoxylin and eosin staining (×400) and quantification of the intima/media ratio were performed on femoral arteries harvested 28 days after wire injury. The intima/media ratio was significantly reduced in the mice treated with GBE compared to the control animals; however, this effect was not seen in the mice treated with both GBE and ZnppIX. Data are expressed as mean ± SEM; n = 6. Scale bars represent 50 μm. *P < 0.05 compared with the control group; ^#^P < 0.05 vs. the GBE-treated group. (n = 6 in each group).

To investigate the mobilization of EPC-like cells into circulation after wire injury, further mice experiments were performed. The number of Sca-1 + /Flk-1 + cells in murine peripheral blood was determined by flow cytometry. As shown in previous studies, mobilization of EPCs contributes to postnatal vascular repair, and this can be enhanced by wire injury in mice^32^. Four days after wire injury, mononuclear cells were isolated from the peripheral blood of the mice. The number of Sca-1 + /Flk-1 + cells (indicating circulating EPC-like cells) in the peripheral blood was significantly elevated in response to vascular wire injury (Fig. 2A). Furthermore, there was a significant increase in the number of Sca-1^+^/Flk-1^+^ cells after vascular injury in the mice with GBE treatment than in those without GBE treatment (P < 0.05). However, these effects of GBE treatment on EPC-like cells were not seen in the presence of ZnPPIX, which is a pharmacological HO-1 inhibitor (Fig. 2A), suggesting the potential involvement of HO-1 in the GBE-induced mobilization of EPC-like cells into the circulation after vascular wire injury in mice.

### The inhibitory effects of GBE on vascular neointimal formation in response to wire injury were abolished by ZnPPIX

Wire injury-induced vascular intimal hyperplasia, as indicated by the intima/media ratio, was reduced in the mice with GBE treatment compared to those without GBE treatment. These effects of GBE were abolished in the presence of ZnPPIX (Fig. 2B), suggesting the potential contribution of HO-1 to the suppressive effects of GBE on neointimal hyperplasia after vascular injury.

### GBE increased HO-1 protein expression and enzyme activity, and proliferation and tube-formation of late EPCs

**Figure 3:**
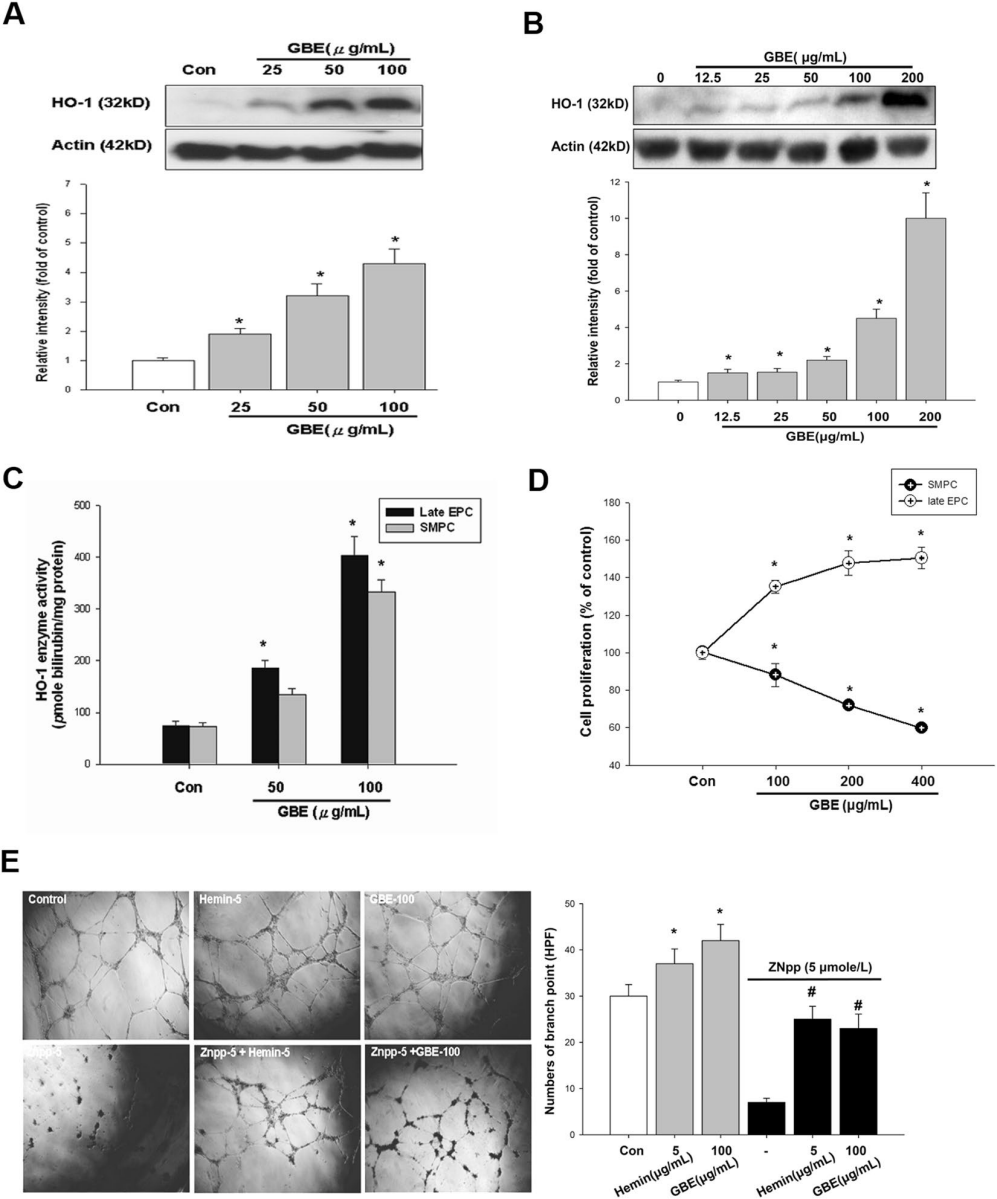
GBE enhanced proliferation, tubule formation, HO-1 expression, and HO-1 enzyme activity in late EPCs and suppressed SMPC proliferation. Late EPCs were incubated with the indicated concentrations of GBE for 24 hours, and HO-1 protein levels and HO-1 enzyme activity were then analyzed. (**A**) A representative immunoblot shows HO-1 and β-actin levels in late EPCs following incubation with the indicated concentrations of GBE for 24 hours. The bar graph shows a summary of the densitometry results of four separate experiments after normalization to β-actin. (**B**) A representative immunoblot shows HO-1 and β-actin protein levels in SMPCs following incubation with an increasing concentration of GBE for 24 hours. The bar graph shows a summary of the densitometry results of four separate experiments after normalization to β-actin. (**C**) Late EPCs and SMPCs were incubated with the indicated concentrations of GBE for 24 hours, and HO-1 activity was then determined by measuring bilirubin production in the cell extracts. (**D**) Proliferation of EPCs and SMPCs was assessed after incubation with the indicated concentrations of GBE for 48 hours. (**E**) Late EPCs in an ECMatrix gel *in vitro* angiogenesis assay. Representative photomicrographs (x100) show inhibition of tube formation in late EPCs in the presence of ZnPPIX (5 μM). The number of endothelial tube junctions in late EPCs treated with GBE and the indicated concentrations of ZnPPIX were quantified. Data are presented as mean ± SEM; n = 6; *P < 0.05 vs. controls.

Human EPCs were cultured and isolated from PBMNCs as previously described [6]. The PBMNCs that were initially seeded on fibronectin-coated wells were round (Supplement Fig. 1). The HO-1 protein levels and HO-1 enzyme activity were determined. Interestingly, after 24 hours of incubation with GBE (50 and 100 μg/mL), the HO-1 expressions were increased by 3.2-fold and 4.3-fold, respectively, in the late EPCs (Fig. 3A). Similar results of HO-1 induction were obtained in the SMPCs, and after 24 hours of incubation with GBE (100 μg/mL), the HO-1 expression was increased by 4.4-fold (Fig. 3B). HO-1 activity was determined by measuring bilirubin production in the cell extracts of late EPCs in response to incubation with the indicated concentrations of GBE and hemin for 24 hours. Incubation with GBE (50 and 100 μg/mL) increased HO-1 activity by 192% and 383%, respectively. These results showed that HO-1 enzyme activity was increased by GBE (Fig. 3C).

Cell proliferation of late EPCs and SMPCs was also assessed. After the EPCs and SMPCs had been incubated with GBE (100 μg/mL) for 2 days, the proliferation rates were determined. GBE (100 μg/mL) increased the proliferation of late EPCs by 43%. Interestingly, under the same conditions, GBE suppressed the proliferation of SMPCs by 17% (Fig. 3D). It has been shown that late EPCs, but not early EPCs, can successfully form a tubular capillary network on Matrigel. [9] An *in vitro* angiogenesis assay was performed with late EPCs to investigate the effects of GBE on EPC neovascularization. After 24 hours of culture, GBE caused a significant 60% increase (P < 0.05) in late EPC tube formation on ECMatrix gel compared to the control group. Quantification of the number of branch points showed a significant reduction in tube formation in the cells treated with ZnPPIX compared to those treated with GBE (Fig. 3E).

### HO-1 induction contributed to the activation of late EPC migration and the suppression of SMPC migration by GBE

**Figure 4:**
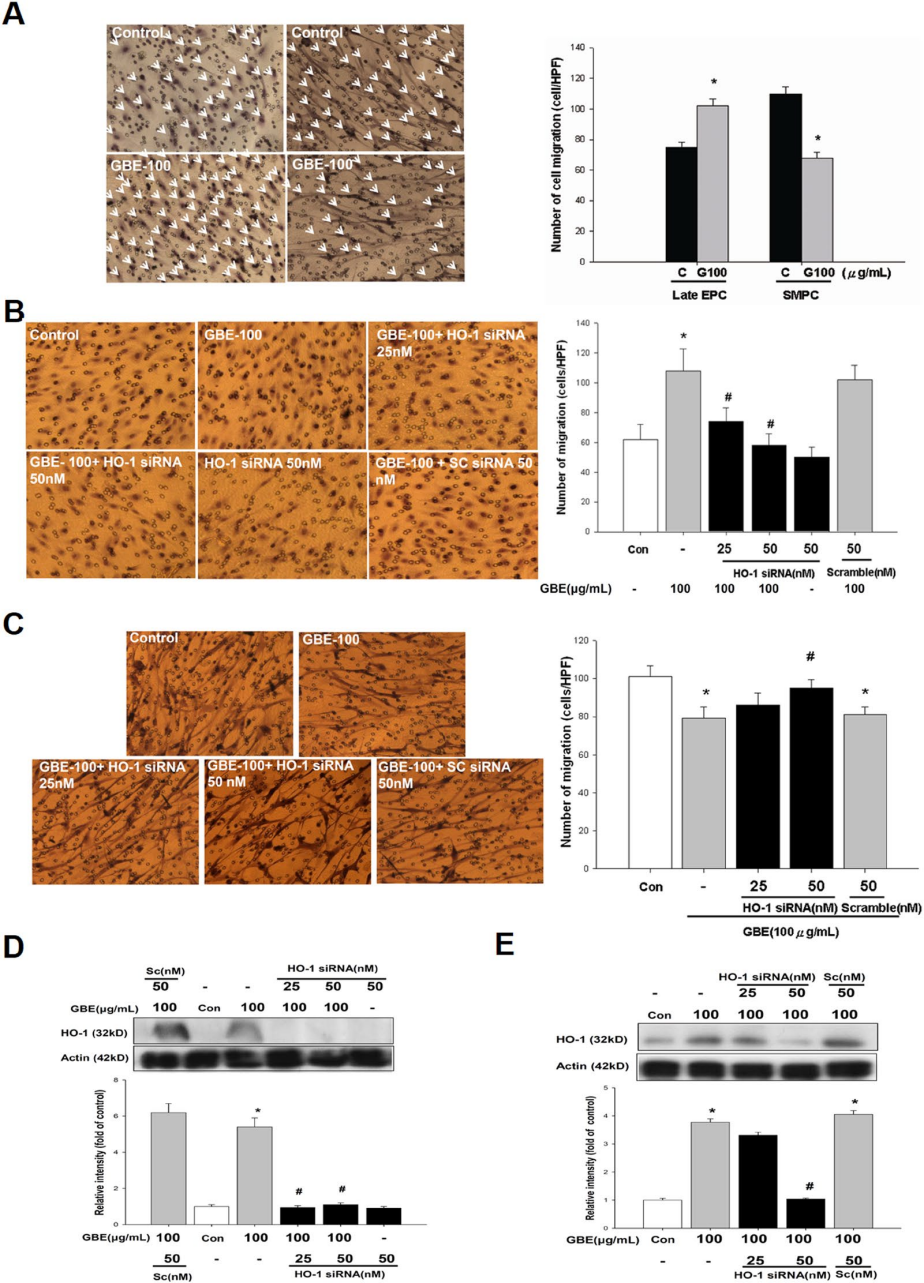
Contribution of HO-1 to GBE-induced activation in the cell migration of late EPCs and suppression of SMPCs. (**A**) EPCs or SMPCs were incubated with the indicated concentrations of GBE for 24 hours, and cell migration was then analyzed using a modified Boyden chamber assay with VEGF as the chemoattractant. In the representative photos, the small dots are holes in the barrier membrane. The migrated cells were stained with hematoxylin and counted under a microscope (x100). (**B**) After incubation of EPCs with the indicated concentrations of GBE and HO-1 siRNA for 24 hours, a modified Boyden chamber assay was used to assess the migratory function of late EPCs. (**C**) After incubation of SMPCs with the indicated concentrations of GBE and HO-1 siRNA for 24 hours, a modified Boyden chamber assay was used to assess the migratory function of SMPCs. (**D**) After incubation of SMPCs with the indicated concentrations of GBE and HO-1 siRNA for 24 hours, HO-1 protein levels were analyzed. A representative immunoblot shows HO-1 and β-actin protein levels in late EPCs following incubation with the indicated concentrations of HO-1 siRNA and GBE for 24 hours. (**E**) A representative immunoblot shows HO-1 and β-actin protein levels in SMPCs following incubation with the indicated concentrations of HO-1 siRNA and GBE for 24 hours. Data are presented as mean ± SEM; n = 6; *P < 0.05 vs. controls; ^#^P < 0.05 vs. the GBE-treated group.

The migratory function of EPCs in response to VEGF is important during neovascularization, and the migratory capacity of late EPCs has been shown to be superior to that of early EPCs *in vitro*^14^. Cell migration was analyzed using a modified Boyden chamber assay with VEGF as a chemoattractant after incubation of the EPCs and SMPCs with different concentrations of GBE for 24 hours. Migration of late EPCs was significantly increased by GBE. Twenty-four hours of incubation with GBE (100 μg/mL) significantly increased late EPC migration by 46%, but decreased SMPC migration by 39.5% (Fig. 4A). These results provided *in vitro* evidence that cultivation in the presence of GBE can enhance the migratory and vasculogenesis capabilities of late EPCs. The migration and homing of EPCs to sites of injury are important early processes in angiogenesis and vascular repair. To investigate whether HO-1 is essential for GBE-induced late EPC migration, Transwell filters were used to determine the number of late EPCs migrating in response to GBE (100 μg/mL) in the presence or absence of various concentrations of HO-1 siRNA. The results indicated that the GBE-induced HO-1 protein expression and late EPC migration were blocked by HO-1 siRNA (Fig. 4B and D). However, the opposite results were observed for SMPCs (Fig. 4C and E). The GBE-attenuated SMPC migration was abolished by HO-1 siRNA. These findings suggested that HO-1 plays a dual role in GBE-induced late EPC migration and GBE-impaired SMPC migration.

### Akt and eNOS phosphorylation were required for the induction of HO-1 by GBE in late EPCs

**Figure 5:**
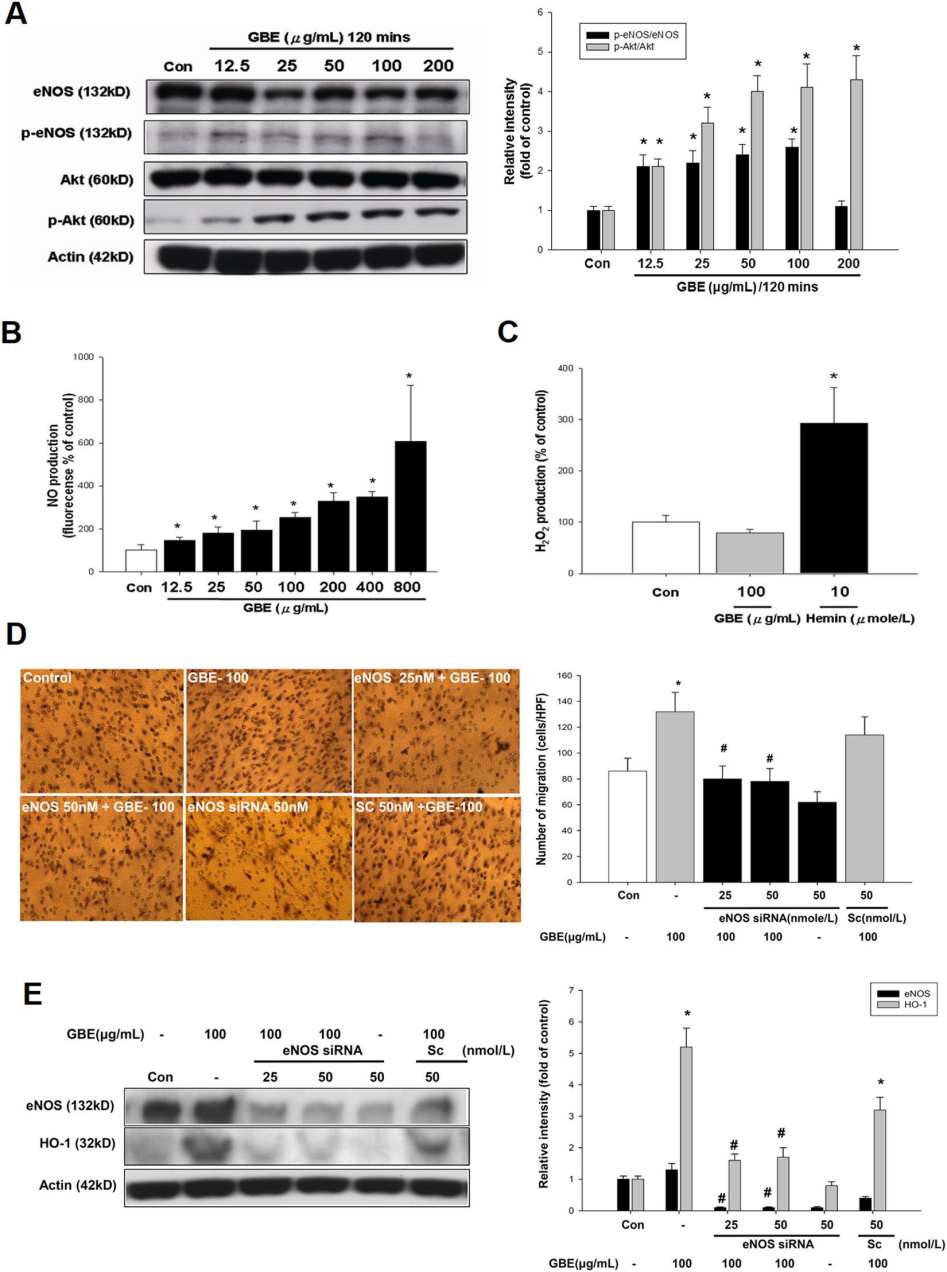
Role of eNOS signaling in GBE-stimulated HO-1 expression. (**A**) After incubation of EPCs with the indicated concentrations of GBE for 2 hours, protein levels of phosphorylated eNOS and NO formation were determined. A representative immunoblot shows the protein levels of phosphorylated-eNOS, eNOS, phosphorylated Akt, Akt, and β-actin in late EPCs in response to GBE. The bar graph shows a summary of the densitometry results of four separate experiments after normalization to β-actin. (**B**) NO production in response to incubation with increasing concentrations of GBE for 3 hours was assessed in late EPCs using DAF-FM probe staining. (**C**) H2O2 production in response to incubation with GBE or hemin for 3 hours was assessed in late EPCs using DCFH-DA probe staining. (**D**) After incubation of EPCs with the indicated concentrations of GBE and eNOS siRNA for 24 hours, a modified Boyden chamber assay was used to assess the migratory function of late EPCs. (**E**) After incubation of EPCs with the indicated concentrations of GBE and eNOS siRNA for 24 hours, eNOS and HO-1 protein levels were analyzed. A representative immunoblot shows eNOS, HO-1, and β-actin protein levels in late EPCs following incubation with the indicated concentrations of eNOS siRNA and GBE for 24 hours. Data are presented as mean ± SEM; n = 6; *P < 0.05 vs. controls; ^#^P < 0.05 vs. the GBE-treated group.

eNOS activity by posttranslational modification at the Akt site has been shown to play a role in the function of EPCs^37^. Therefore, this study investigated the effects of GBE on NO signaling in EPCs. After incubation of late EPCs with GBE (50 and 100 μg/mL) for 2 hours, Western blotting showed that Akt phosphorylation at Ser^473^ and eNOS phosphorylation at Ser^1177^ were significantly increased (by 112% and 132%, respectively) compared with cells cultured in control media (Fig. 5A). This activation of eNOS phosphorylation was associated with an increase in late EPC-derived intracellular NO production (175% increase with 100 μg/mL GBE) (Fig. 5B) and a minimal alteration in total eNOS expression, suggesting the selective activation of eNOS by GBE. In addition, incubation with GBE for 3 hours did not alter the production of ROS, as indicated by the level of H2O2; however, incubation with heme significantly increased ROS production in late EPCs, suggesting that the effects of GBE on EPCs may be independent of ROS (Fig. 5C). We then investigated whether eNOS plays a role in the function of late EPCs. After transfection of late EPCs with increasing concentrations of eNOS siRNA followed by incubation with GBE for 24 hours, the expression of eNOS and cell migration were dramatically reduced (Fig. 5D,E). GBE-stimulated HO-1 accumulation was also attenuated by eNOS siRNA, suggesting that HO-1 induction by GBE may require eNOS.

### GBE increased HO-1 expression, differentiation, adhesion, and proliferation ability in early EPCs

**Figure 6:**
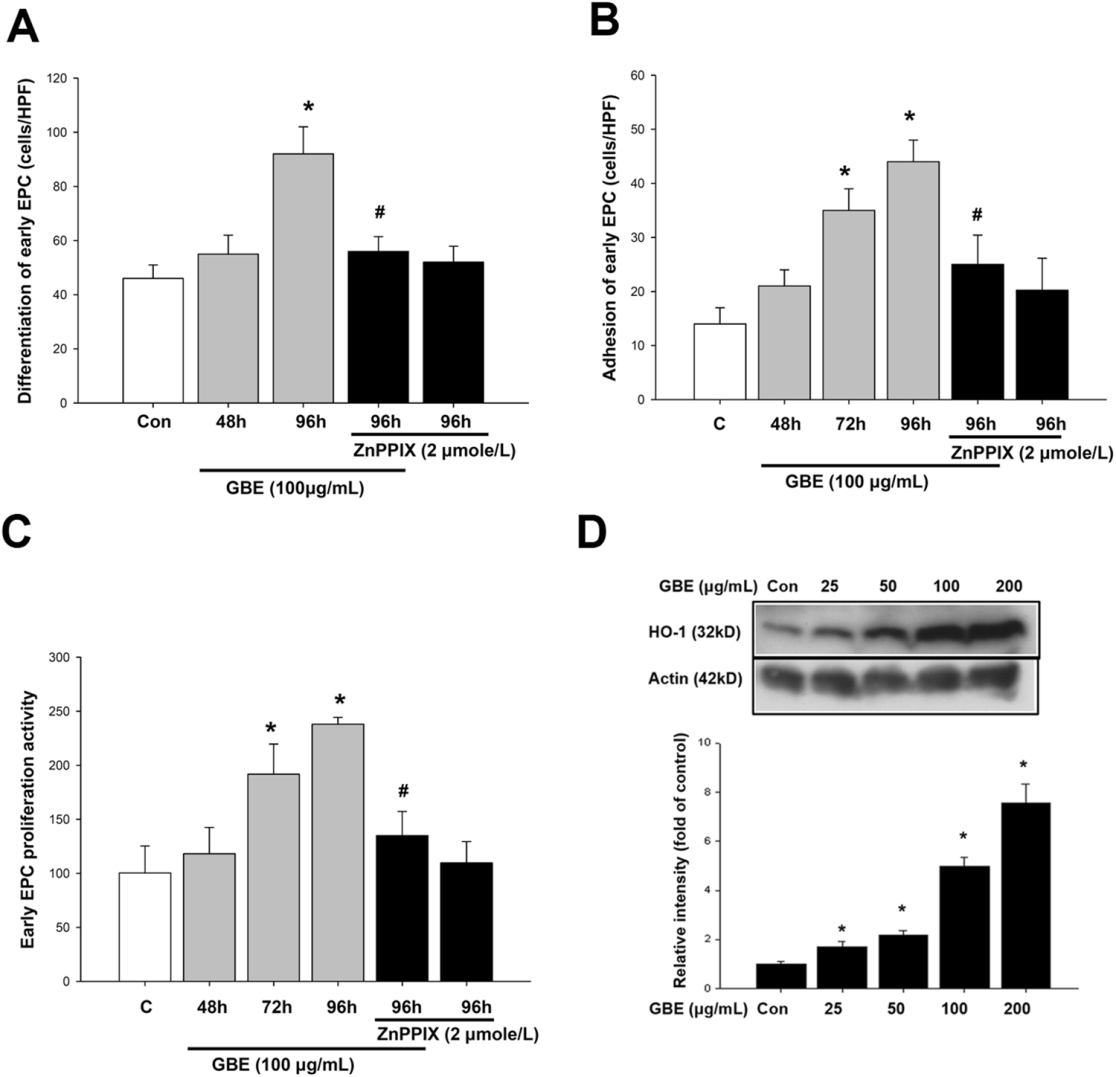
GBE increased the number, adhesion, migration, proliferation, and HO-1 expression in early EPCs. After seeding MNCs, the cells were incubated with GBE for different time periods. GBE exposure increased the number of adherent early EPCs in a time-dependent manner, as assessed by DiI-ac-LDL staining (P < 0.05). (**A**) Incubation of MNCs with GBE (100 μg/mL) for 4 days (96 hours) increased cell differentiation and early EPCs, as assessed by DiI-ac-LDL staining. The effects of GBE were inhibited in the presence of ZnPPIX (2 μmole/L) (**B**) Incubation of early EPCs with GBE (100 μg/mL) for 3 or 4 days (72 or 96 hours) increased cell adherence to fibronectin-coated plates and cell migration in response to VEGF. The effects of GBE were inhibited in the presence of ZnPPIX (2 μmole/L). (**C**) Incubation of early EPCs with GBE (100 μg/mL) for 3 or 4 days (72 or 96 hours) increased cell proliferation. The effects of GBE were inhibited in the presence of ZnPPIX (2 μmole/L). (**D**) Incubation of early EPCs with increasing concentrations of GBE (25, 50, 100, and 200 μg/mL) for 24 hours dose-dependently increased HO-1 protein levels, as assessed by Western blotting.

Human EPCs were cultured and isolated from PBMNCs as previously described^8^. The PBMNCs that were initially seeded on fibronectin-coated wells were round (Supplement Fig. 1). After seeding the PBMNCs into wells, the cells were incubated with a fixed concentration of GBE for 2 or 4 days. Incubation of the cells with GBE increased the number of differentiated, adherent early EPCs in a time-dependent manner. Compared with the control conditions, incubation with GBE (100 μg/mL) for 2 or 4 days increased the number of early EPCs positive for DiI-ac-LDL uptake by 19.5% and 101%, respectively (P < 0.05) (Fig. 6A). Adhesion assays were also performed to evaluate the vascular repair activity and capacity of EPCs^33,34^. Early EPCs were incubated with GBE (100 μg/mL) for 2, 3, and 4 days respectively. The attachment of early EPCs to fibronectin-coated plates was significantly increased by 250% in 72 hours and 314% in 96 hours, respectively (Fig. 6B). EPC proliferation was assessed using the MTT assay. After incubation of early EPCs with GBE (100 μg/mL) for the indicated times, proliferation was increased by 191% after 3 days of incubation (Fig. 6C). Early EPCs were incubated with varying concentrations of GBE (25, 50, 100, and 200 μg/mL) for 24 hours, and HO-1 protein levels were evaluated by Western blot. Incubation with GBE (100 and 200 μg/mL) significantly increased the HO-1 protein expression by 4.9-folds (with GBE 100 μg/mL) and 7.5-folds (with GBE 200 μg/mL), respectively (Fig. 6D). However, in the presence of ZnPPIX (2 μmole/L), GBE-mediated early EPC differentiation, adhesion, and proliferation were abolished individually (Fig. 6A,B,C). These results suggested that HO-1 was involved the GBE-mediated upregulation of early EPC function.

### Phosphoinositide 3-kinase (PI3K) and mitogen-activated protein kinase (MAPK) signaling pathways differentially contributed to GBE-stimulated HO-1 expression in late EPCs and SMPCs

**Figure 7:**
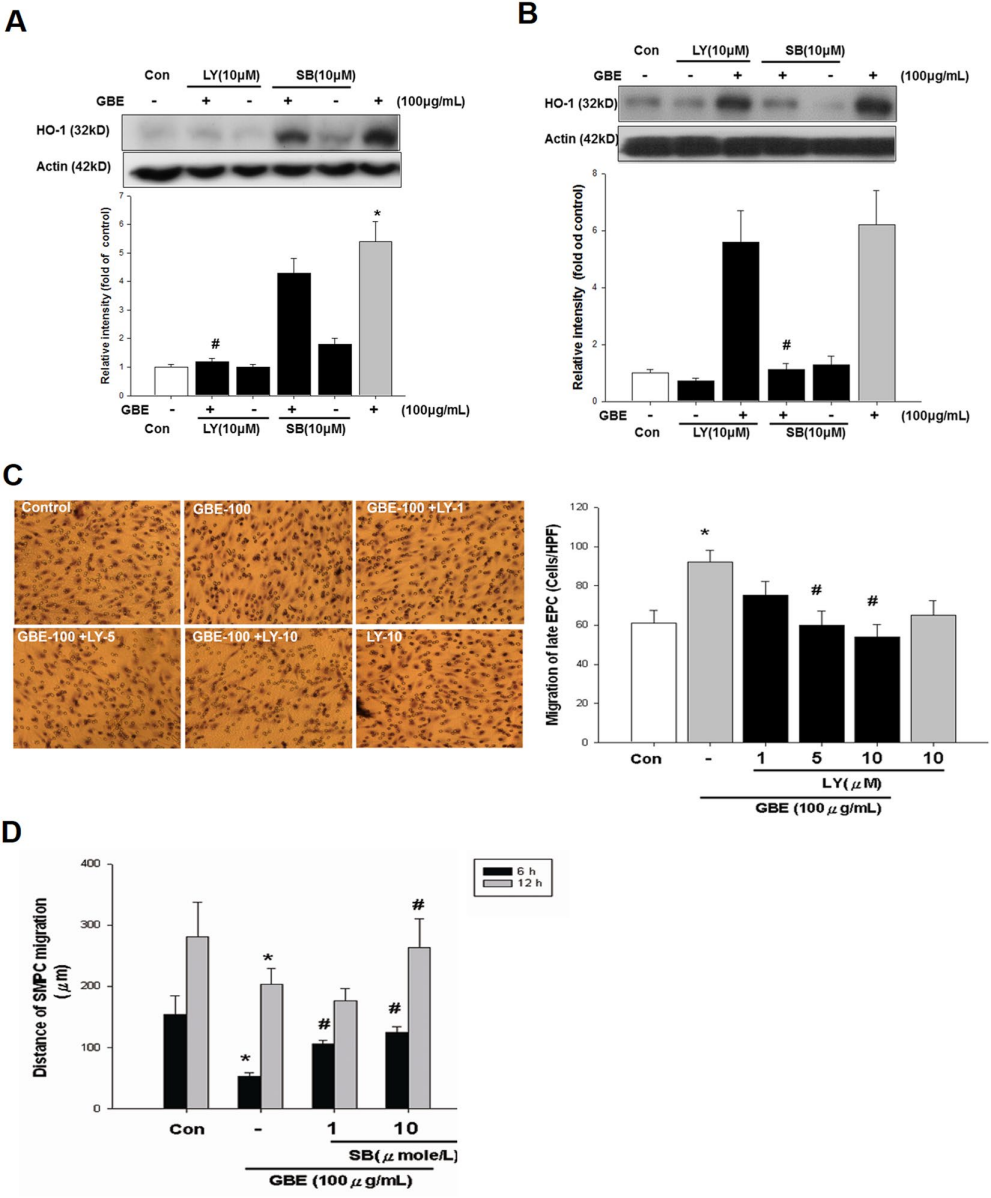
Role of PI3K and p38 MAPK signaling in GBE-stimulated HO-1 protein accumulation. (**A**) A representative immunoblot shows HO-1 protein levels in late EPCs in response to treatment with LY294002 (PI3K inhibitor) or SB203580 (p38 MAPK inhibitor) for 30 minutes, followed by incubation with GBE for 24 hours. (**B**) A representative immunoblot shows HO-1 protein levels in SMPCs in response to treatment with various concentrations of LY294002 (PI3K/Akt inhibitor) or SB203580 (p38 MAPK inhibitor) for 30 minutes, followed by incubation with GBE for 24 hours. (**C**) Late EPCs in a modified Boyden chamber assay. Representative photomicrographs show LY294002 (10 μM) inhibition of the migration of late EPCs. Quantification of the number of late EPCs that migrated after GBE treatment; the indicated concentrations of LY294002 are shown below. (**D**) SMPCs in a wound healing migration assay. Quantification of the distance of migration in SMPCs treated with or without GBE and SB203580 was shown. GBE treatment for 6 or 12 hours significantly inhibited the migration of SMPCs as compared with controls, which could be reversed by co-incubation with SB203580 (1 or 10 μM) in a dose-dependent manner. Data are presented as mean ± SEM; n = 6; *P < 0.05 vs. controls; ^#^P < 0.05 vs. the GBE-treated group.

We next investigated the effect of phosphoinositide 3-kinase (PI3K)/Akt and mitogen-activated protein kinase (MAPK) activity on the GBE-induced HO-1 expression in late EPCs and SMPCs. Cells were incubated for 30 minutes with the PI3K inhibitor LY294002 (10 μM), p38 MAPK inhibitor SB203580 (10 μM), and PD98059 or SP600125 (data not shown), followed by incubation with GBE (100 μg/mL) for 24 hours. The HO-1 protein expression in the late EPCs, as shown by Western blot analysis, was significantly decreased with LY294002 treatment compared with GBE treatment alone (Fig. 7A). However, HO-1 protein accumulation in the SMPCs was significantly decreased with SB203580 treatment compared with GBE treatment alone (Fig. 7B). Treatment of the late EPCs with GBE followed by incubation with LY294002 (10 μM) resulted in considerable inhibition of GBE-induced EPC migration compared with the controls. GBE stimulated the migration of late EPCs, suggesting that HO-1 plays a crucial role in the function of EPCs. Quantification of the number of migrated cells showed a significant reduction in the migrated cells treated with LY294002 compared to those treated with GBE (Fig. 7C). Treatment of the SMPCs with GBE followed by incubation with SB203580 (10 μM) resulted in considerable inhibition of GBE-induced SMPC migration compared with the controls in the *in vitro* wound healing model. GBE stimulated the migration of SMPCs, suggesting that HO-1 plays a crucial role in the function of SMPCs (Fig. 7D). These results indicated that GBE may upregulate late EPCs through modulating PI3K/Akt- and p38-related mechanisms in SMPCs.

## Discussion

There are several major findings in this study. First, GBE treatment suppressed wire injury-induced vascular neointimal hyperplasia with an increase in bone marrow-derived EPC mobilization and homing, and reduced the mobilization and homing of SMPCs to the site of injury. Mechanistic investigations showed that co-treatment with ZnPPIX, which is an HO-1 inhibitor, abolished the beneficial effects of GBE, suggesting that GBE-induced HO-1 played a crucial role in vascular repair/remodeling in this animal model. Second, long-term *in vitro* exposure to GBE activated early and late EPCs, and impaired the migration of SMPCs alone with a universal increase in the expression of HO-1 in these three types of cells, suggesting the dual role of GBE-induced HO-1 in EPCs and SMPCs individually. Third, GBE induced the expression of HO-1 differentially through PI3K/AKT/eNOS pathways in the late EPCs and via p38-related mechanisms in the SMPCs. Taken together, these results suggest that HO-1 induction, regardless of the mechanism, plays a critical and complex role in the beneficial effects of GBE on vascular repair after wire injury. Our findings may add a new rationale for the future validation of the pharmacological induction of HO-1 as a potential therapeutic strategy for vascular repair and clinical atherosclerotic diseases.

The results of the current study are in line with our previous findings, in that a long guanidine-thymidine dinucleotide repeat ([GT]n; with n ≥ 30) located in the HO-1 promoter and involved in the expression of HO-1 is associated with increased restenosis after angioplasty^38^. The efficacy of HO-1 gene transfer has been shown in several animal models of cardiovascular disease, including restenosis after angioplasty and atherosclerosis^39,40^. HO-1 induction by probucol has also recently been shown to activate EPCs and promote reendothelialization in areas distant to the intact endothelium^41,42^. The present study also showed that HO-1 induced by GBE not only enhanced the activity of early and late EPCs, but also inhibited the migration of SMPCs, suggesting the novel and complex role of HO-1 induction in modulating various vascular progenitor cells including EPCs and SMPCs for vascular repair.

Denudation of the endothelium, as caused by wire injury, combined with a rapid increase in leukocyte infiltration leads to increased SMC proliferation and formation of neointima. The loss of the endothelium is perhaps the initiating element associated with subsequent restenosis. The direct effects of HO-1 on inducing the growth arrest of VSMCs and preventing neointimal expansion *in vivo* are clear^39^. The present *in vitro* and *in vivo* observations, including recruitment of bone marrow progenitor cells to the site of injury, support the concept that GBE fosters earlier reendothelialization and is involved in the differentiation and motility of EPCs in an effort to augment the repair of the injured vessels, which ultimately contributes to less intimal hyperplasia. In this study, GBE induced vascular progenitor cells to generate HO-1, and this may ultimately have led to restoration of normal artery and vessel size. These results agree in part with those of a previous study, which demonstrated that HO-1-deficient mice were unable to form capillary sprouts in ischemia-induced angiogenesis^20,43^.

Our *in vitro* results also suggest that HO-1, as induced by a pharmacologic approach, could enhance cell migration and tube formation in human microvascular endothelial cells and EPCs via VEGF. Previous studies have shown that HO-1 is involved in ischemia-induced angiogenesis^20,21,44^. In addition, a recent *in vitro* study showed that the vascular repair properties of HO-1 can be attributed to carbon monoxide (CO) production^20^. CO is an established cellular messenger with signaling functions similar to NO, and it has been shown to cause redistribution and phosphorylation of a cytoskeleton-associated protein (vasodilator-stimulated phosphoprotein), which is involved in EPC migration. In the present study, HO-1 induced by GBE increased VEGF-augmented migration of late EPCs through eNOS dependent mechanisms. Given that GBE could also directly activate eNOS in the late EPCs, it is likely that both NO, at least in part, and CO contributed to the beneficial effects of GBE on vascular repair in this animal model.

The three established types of vascular progenitor cells are early EPCs, late EPCs, and SMPCs, which are classified within *in vitro* culture systems according to their time-dependent appearance^7,11^. In the pathogenesis of vascular diseases, a reduced number of EPCs has been reported to delay vascular repair^45,46^. Therefore, acceleration of the function of late EPCs along with the suppression of the function of SMPCs may contribute to vascular healing and lesion prevention in response to vascular injury. In the present study, we demonstrate for the first time the differential effects of GBE on cell proliferation/migration in early and late EPCs and SMPCs, indicating the possible universal effects of GBE on different vascular progenitor cells. We also demonstrated the *in vivo* effects of GBE to reduce the attachment of bone marrow-derived SMPCs or VSMC-like cells with increased attachment of bone marrow-derived EPCs or endothelial-like cells at the vascular injury sites. Both the enhancement of EPCs and suppression of SMPCs could be attributed to the induction of HO-1 by GBE, which may be of particular clinical interest for complex vascular repair after mechanical injury, such as that in percutaneous coronary interventions.

Stem cells and progenitor cells are highly potent regenerative cells with high proliferative capacity^47^. In the present study, long-term incubation with GBE increased the proliferation of both early and late EPCs, but suppressed the proliferation of SMPCs. Exposure to LY294002, which is a PI3K/Akt inhibitor, significantly reduced the number and proliferation of both monocytes and early EPCs (data not shown). In late EPCs, GBE-stimulated HO-1 accumulation was significantly reduced by co-incubation with LY294002 or eNOS siRNA. Taken together, GBE may induce HO-1 through PI3K/Akt/eNOS mechanisms in both early and late EPCs. Such mechanisms may then contribute to both the differentiation of early EPCs and the activation of late EPCs. Our findings are consistent with a previous report in that GBE 761 may increase *in vitro* and *in vivo* endothelial eNOS expressions^48^. Furthermore, eNOS activation was critical to HO-1 induction by GBE in this study, suggesting that adequate interactions between NO- and CO-activation systems are required for the activation of late EPCs. Future studies are warranted to clarify whether the direct supplementation of NO by NO donors can activate HO-1 in vascular progenitor cells.

On the other hand, the GBE-induced HO-1 expression may have been independent of oxidative stress in this study. First, blockade of the generation of ROS did not affect the GBE-induced HO-1 expression in different vascular progenitor cells (data not showed). Second, while ROS generation was observed with hemin treatment (data not shown), 3 hours of exposure to GBE did not increase ROS generation, suggesting that the mechanism of HO-1 induction by GBE is different from that by hemin in late EPCs.

Previous reports have shown that HO-1 can suppress SMC proliferation and stimulate apoptosis, possibly through p53-related mechanisms^49,50^. In this study, GBE induced the expression of HO-1 and reduced the proliferation and migration of SMPCs, and this could be blocked by the presence of SB2003580, which is a p38 inhibitor, but not by LY294002, which is a PI3K/Akt inhibitor. Accordingly, p38 pathways were involved in the GBE-induced HO-1 expression in SMPCs but not in EPCs. However, we also found that, in the absence of GBE, SB2003580 alone could induce the expression of HO-1 and suppress the migration of SMPCs to some extent. One possible explanation is that activation of p38 pathways is essential in SMPCs in addition to HO-1 induction. Several other signal pathways are complementary to and competitive with p38 pathways, and they may also contribute to HO-1 induction and could be activated if the major p38 pathway is blocked. While p38 pathways played a major role in the GBE-induced HO-1 expression in the SMPCs in this study, future studies are still required to clarify the complex mechanisms and the interesting effects of HO-1 induction in SMPCs. These novel findings support the complex and universal role of HO-1 in modulating different vascular progenitor cells for vascular repair.

There are some concerns about the HO-1 induction by GBE in this study. First, GBE is available as a standardized preparation (commercial name: Cerenin^®^), and it is the most widely sold phytomedicine in Europe and the United States, where it is used to treat the symptoms of early-stage Alzheimer’s disease, vascular dementia, peripheral claudicating, and tinnitus of vascular origin. Recently, GBE was shown to inhibit the expression of inflammatory markers such as high sensitive C-reactive protein, tumor necrosis factor-α, and interleukin-6 in patients with metabolic syndrome^51^. Furthermore, GBE was shown to have protective effects on bone marrow mesenchymal stem cells against oxidative stress injury by regulating p38MAPK and JNK signaling^52^. It is not known if the above clinical and *in vitro* effects of GBE could be related to the HO-1 mechanisms. In fact, similar concentrations have been used in other *in vitro* studies in which GBE was shown to have a protective effect in neurons and human lung endothelial cells dependent on HO-1^35,53^. However, given the complex and multiple pathways involved in the induction of HO-1, it is not known whether the effects of GBE on different vascular progenitor cells, as observed in this study, would also be seen with other HO-1 inducers such as cytokines. Second, commercial purveyors of Ginkgo biloba tablets recommend a daily dose of 200 mg^54^. Thus, a single dose of commercial GBE would not reach the levels of extract used in the present study. However, in our *in vivo* mouse study, both the accumulation of HO-1 and enzyme activity in circulating mononuclear cells were increased by GBE treatment (100 mg·kg^−1^·day^−1^) (data not shown), suggesting the potential cumulative effects of GBE *in vivo*. In addition to our findings, another study reported that hemin-induced HO-1 expression could ameliorate cigarette smoke-related restenosis after vascular injury in an animal model through the inhibition of inflammatory cytokines and adhesion molecules. In addition, HO-1 gene promoter polymorphisms have been shown to be associated with coronary artery disease and restenosis after percutaneous coronary interventions in a meta-analysis.

## Conclusions

In summary, GBE treatment improved vascular repair after mechanical injury with an increase in EPC and reduction in SMPC accumulation at the sites of injury in this study (Supplement Fig. 2). These effects could be blocked by ZnPPIX, an HO-1 inhibitor. *In vitro* studies showed that HO-1 was required for the GBE-induced upregulation of EPCs and downregulation of SMPCs. GBE induced the expression of HO-1 by activating Akt/eNOS signaling in EPCs and p38 pathways in SMPCs. Furthermore, both HO-1 and eNOS siRNA impaired the GBE-induced migration of late EPCs, suggesting the complimentary roles of NO- and CO-regulated mechanisms in GBE-induced EPC activation. Accordingly, our findings suggest novel mechanisms of HO-1 with regards to the effects of GBE on different vascular progenitor cells and vascular remodeling in response to mechanical injury, and provide a rationale for the potential role of therapy targeting GBE and HO-1 for vascular protection in clinical atherosclerosis and related cardiovascular diseases. Future clinical studies are indicated to validate the current *in vivo* and *in vitro* findings.

## Supplementary information

Supplementary information

## Data Availability

Supplementary materials can be found at https://jbiomedsci.biomedcentral.com/
