# Supplementary material for: Activation of heme oxygenase-1 by Ginkgo biloba extract differentially modulates endothelial and smooth muscle-like progenitor cells for vascular repair
Source: Sci Rep. 2019 Nov 21;9:17316. doi: 10.1038/s41598-019-53818-7 (PMC6872755; doi:10.1038/s41598-019-53818-7)

Title:

Activation of heme oxygenase-1 by Ginkgo biloba extract differentially modulates endothelial and smooth muscle-like progenitor cells for vascular repair

Authors:

Tao-Cheng Wu, Jia-Shiong Chen, Chao-Hung Wang, Po-Hsun Huang, Feng-Yen Lin, Liang-Yu Lin, Shing-Jong Lin, and Jaw-Wen Chen*

Supplementary Fig. 1

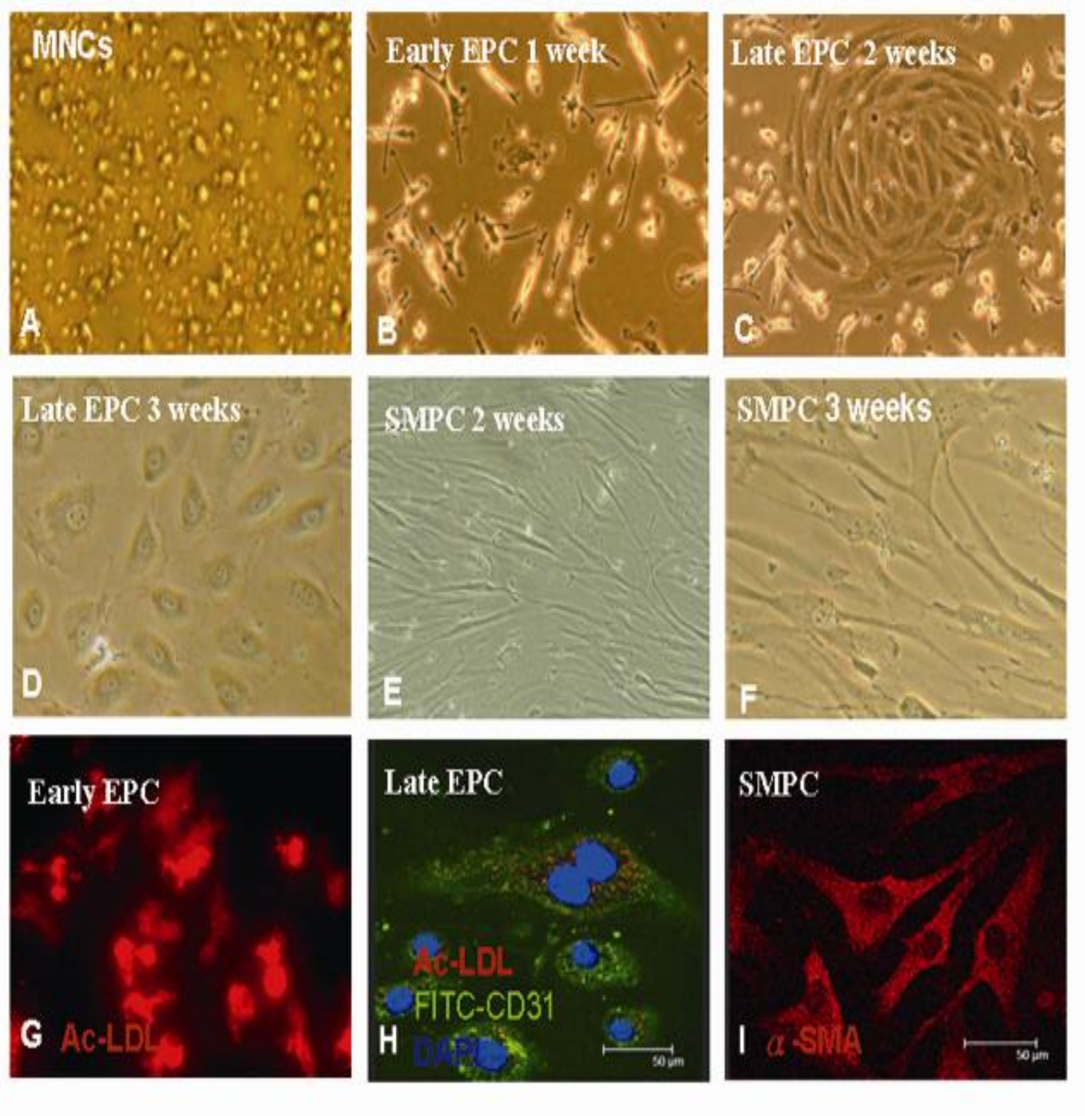

Supplementary Figure 1.

Morphology and characterization of circulation early, late endothelial progenitor cells, and smooth muscle progenitor cells. [A] MNCs were isolated and plated on fibronectin-coated culture dishes on the first day. [B] Four days after plating, adherent circulating early EPCs with spindle shapes were observed. [C] Fourteen days and [D] twenty-one after plating, late EPCs with cobblestone-like morphology were selected and reseeded. [E] Fourteen days and [F] twenty-one after plating, SMPCs with spindle shapes morphology were selected and reseeded, like mature smooth muscle cell. [G] Immunofluorescence detected s DiI-acLDL uptake in early EPCs. [H] Late EPCs were shown to simultaneously endocytose DiI-acLDL (red) and immunofluorescence detected (green) CD31. [I] SMPCs were shown to immunofluorescence detected α-SMA. Scale bar: 50 μm.

Supplementary Fig. 2

A.

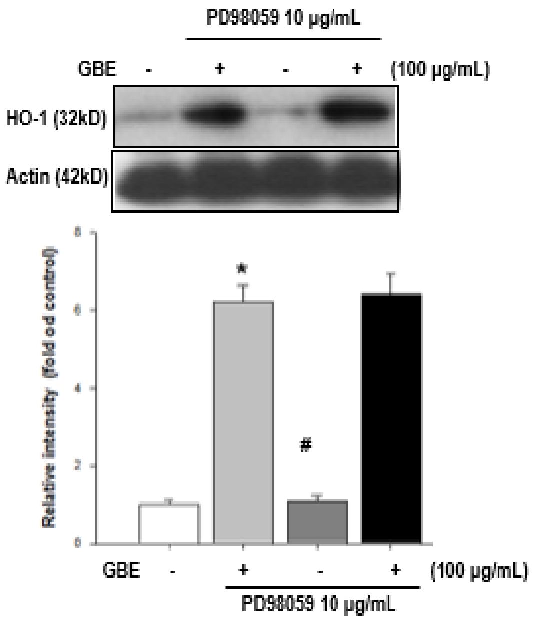

B.

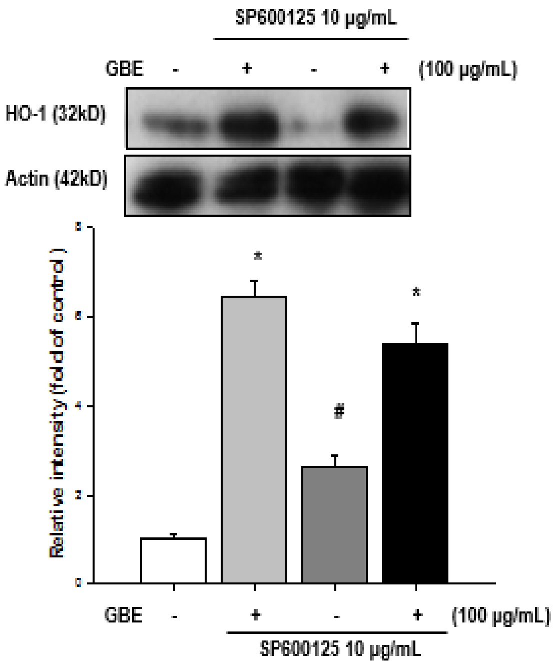

Supplementary Figure 2.

Role of ERK and JNK signaling in GBE-stimulated HO-1 protein accumulation. [A] A representative immunoblot shows HO-1 protein levels in late EPCs in response to treatment with PD98059 (ERK inhibitor) for 30 minutes, followed by incubation with GBE for 24 hours. [B] A representative immunoblot shows HO-1 protein levels in late EPCs in response to treatment with SP600125 (JNK inhibitor) for 30 minutes, followed by incubation with GBE for 24 hours. Data are presented as mean ± SEM; $n = 6$; *$P < 0.05$ vs. controls; #$P < 0.05$ vs. the GBE-treated group.

Fig. 3A

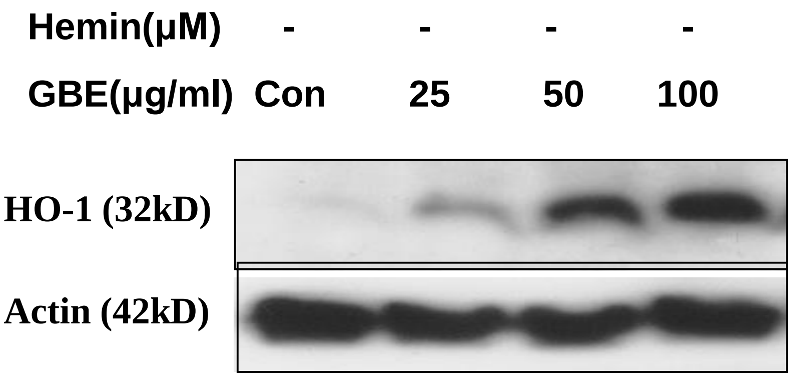

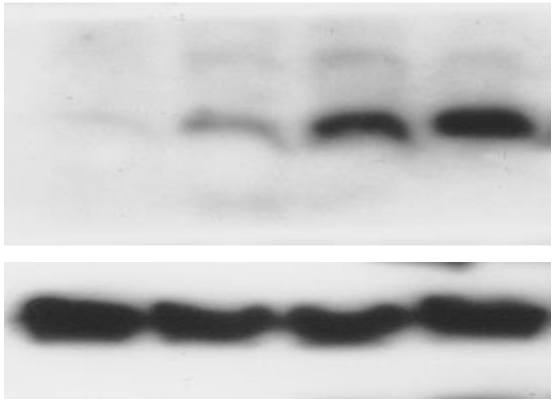

Fig. 3B

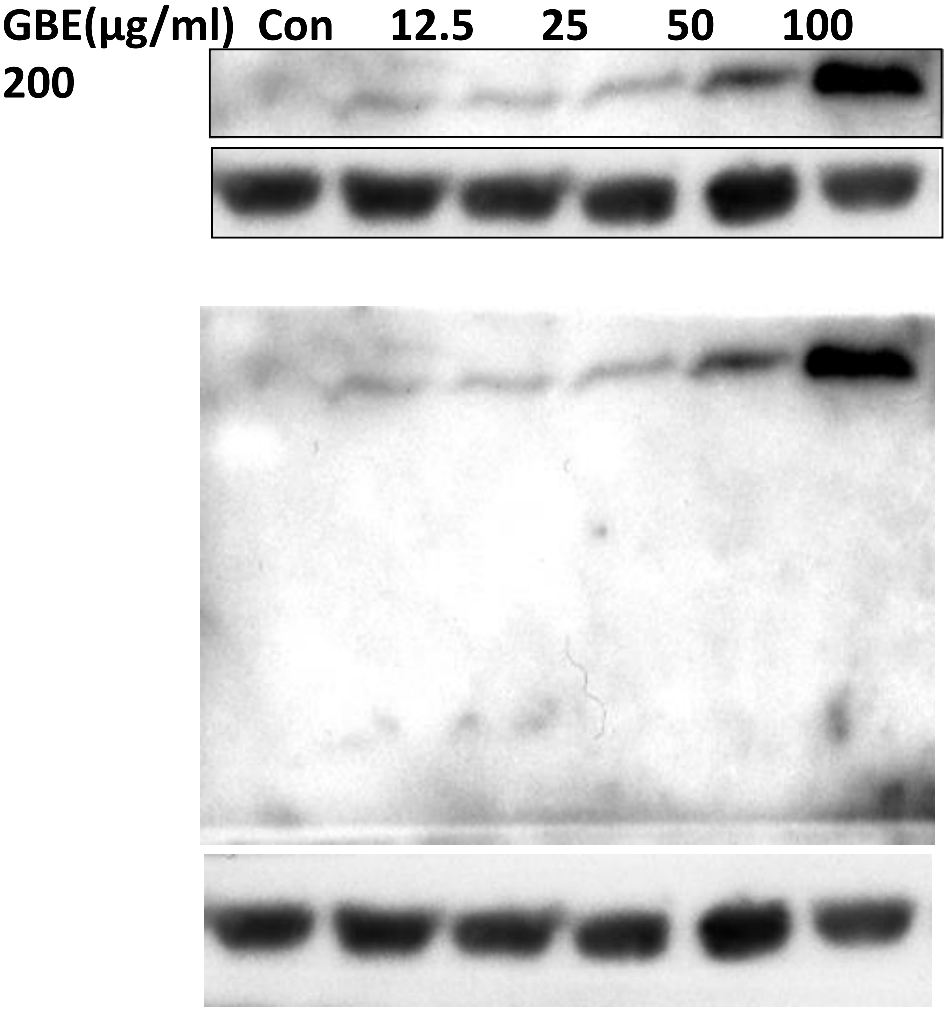

Fig. 4D

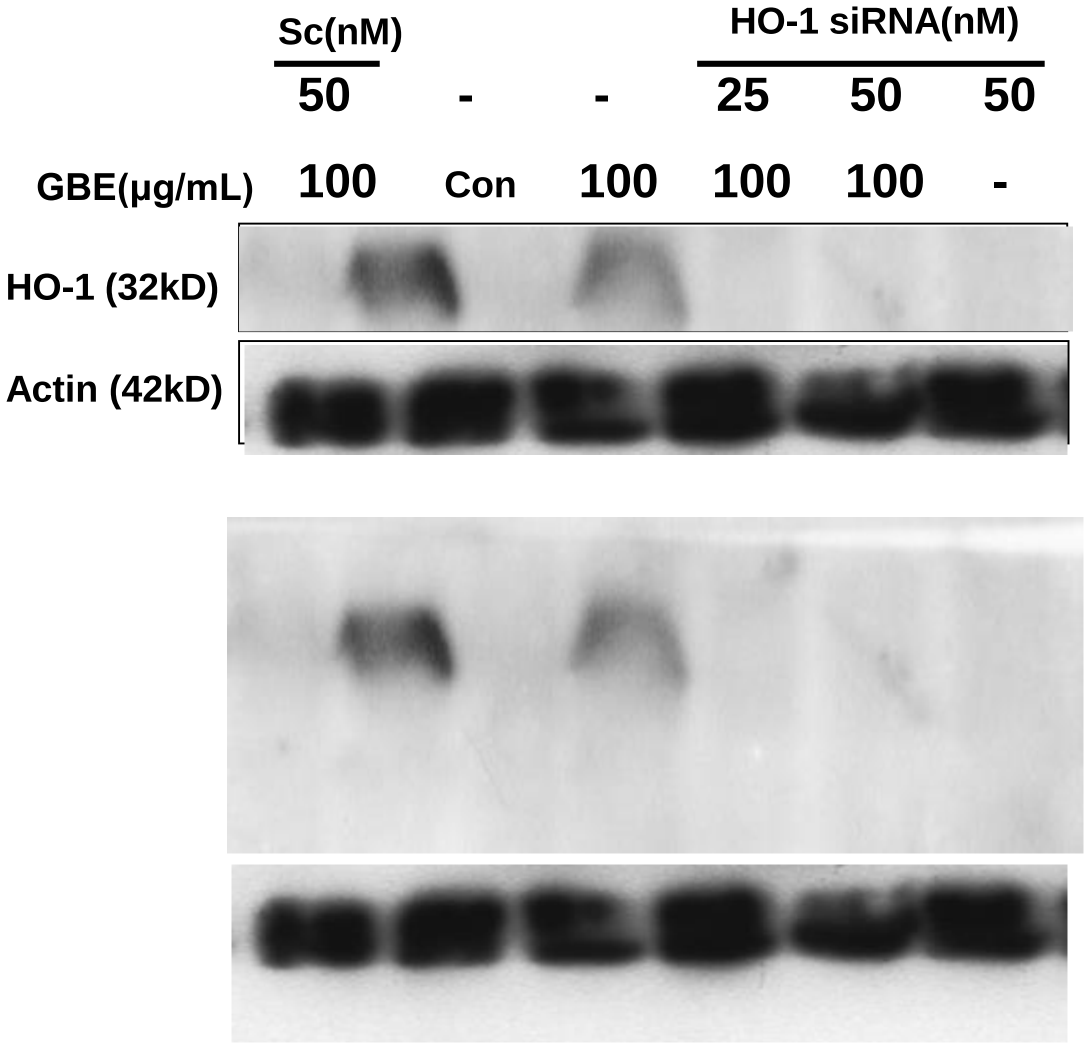
Sc(nM)
HO-1 siRNA(nM)
50 - - 25 50 50
GBE(μg/mL) 100 Con 100 100 100 -
HO-1 (32kD)
Actin (42kD)

Fig. 4E

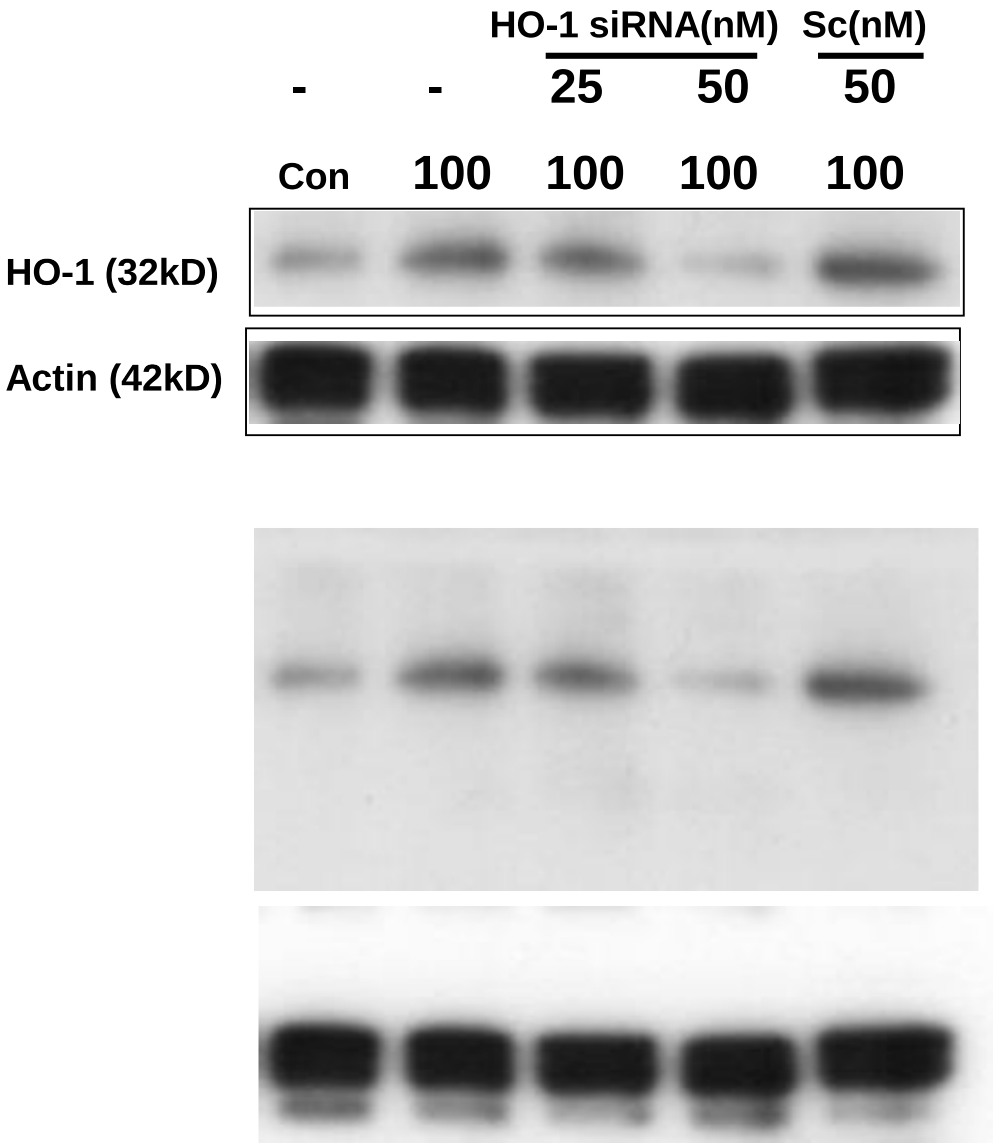

HO-1 siRNA(nM)
Sc(nM)
- - 25 50 50
Con 100 100 100 100
HO-1 (32kD)
Actin (42kD)

Fig. 5A

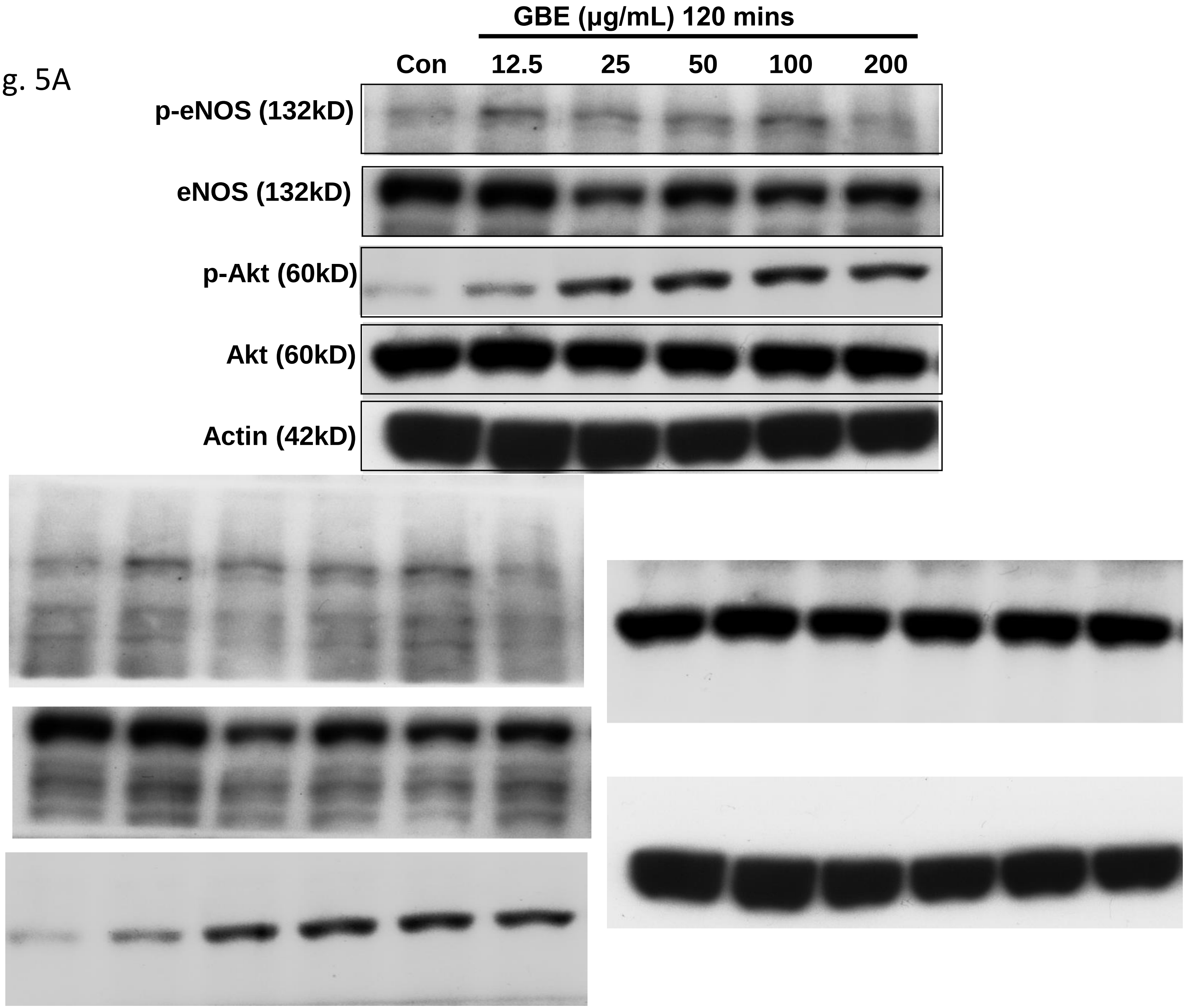
GBE (μg/mL) 120 mins
Con
12.5
25
50
100
200
p-eNOS (132kD)
eNOS (132kD)
p-Akt (60kD)
Akt (60kD)
Actin (42kD)

Fig. 5E

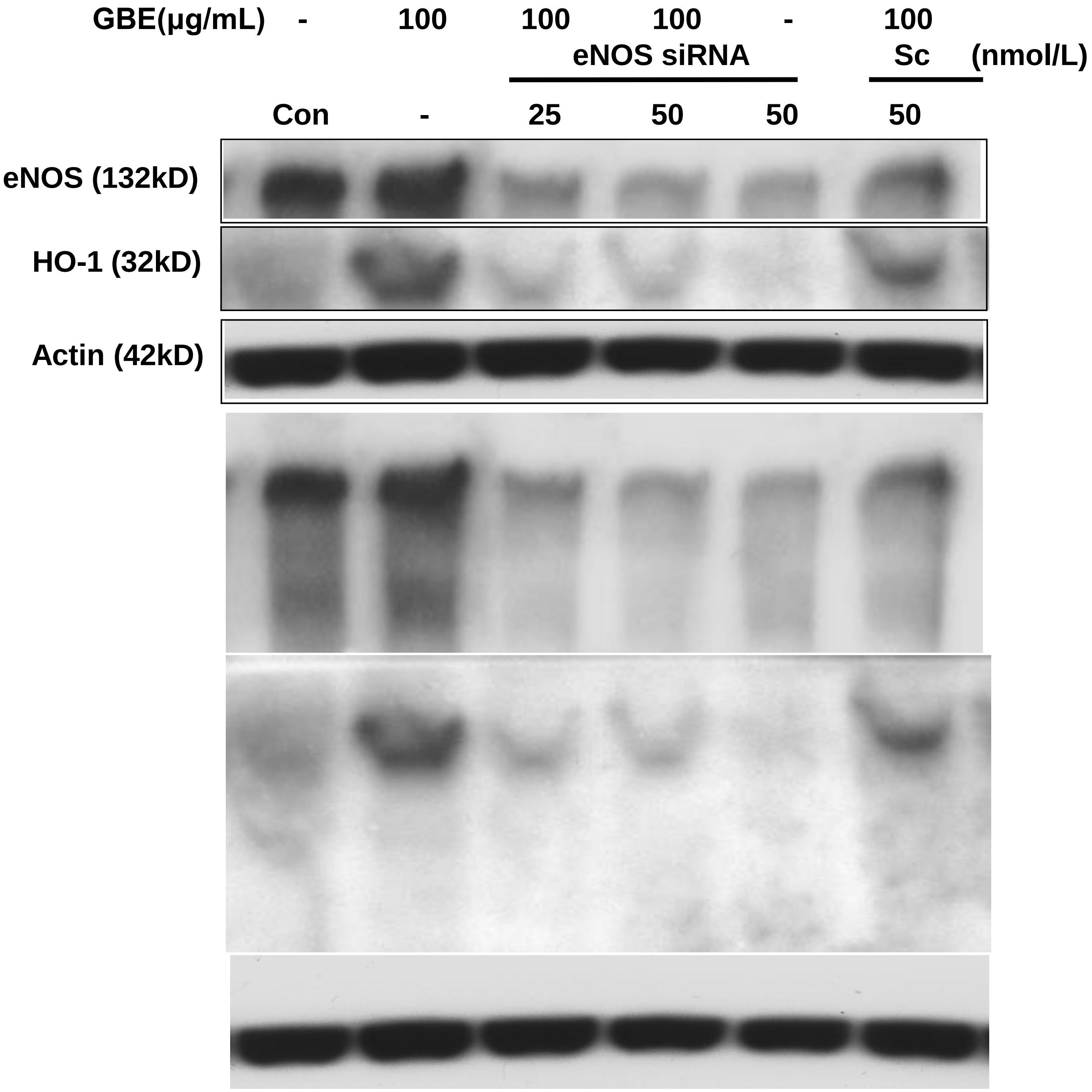

GBE(μg/mL) - 100 100 100 - 100
eNOS siRNA
Sc
(nmol/L)
Con - 25 50 50 50
eNOS (132kD)
HO-1 (32kD)
Actin (42kD)

Fig. 6D

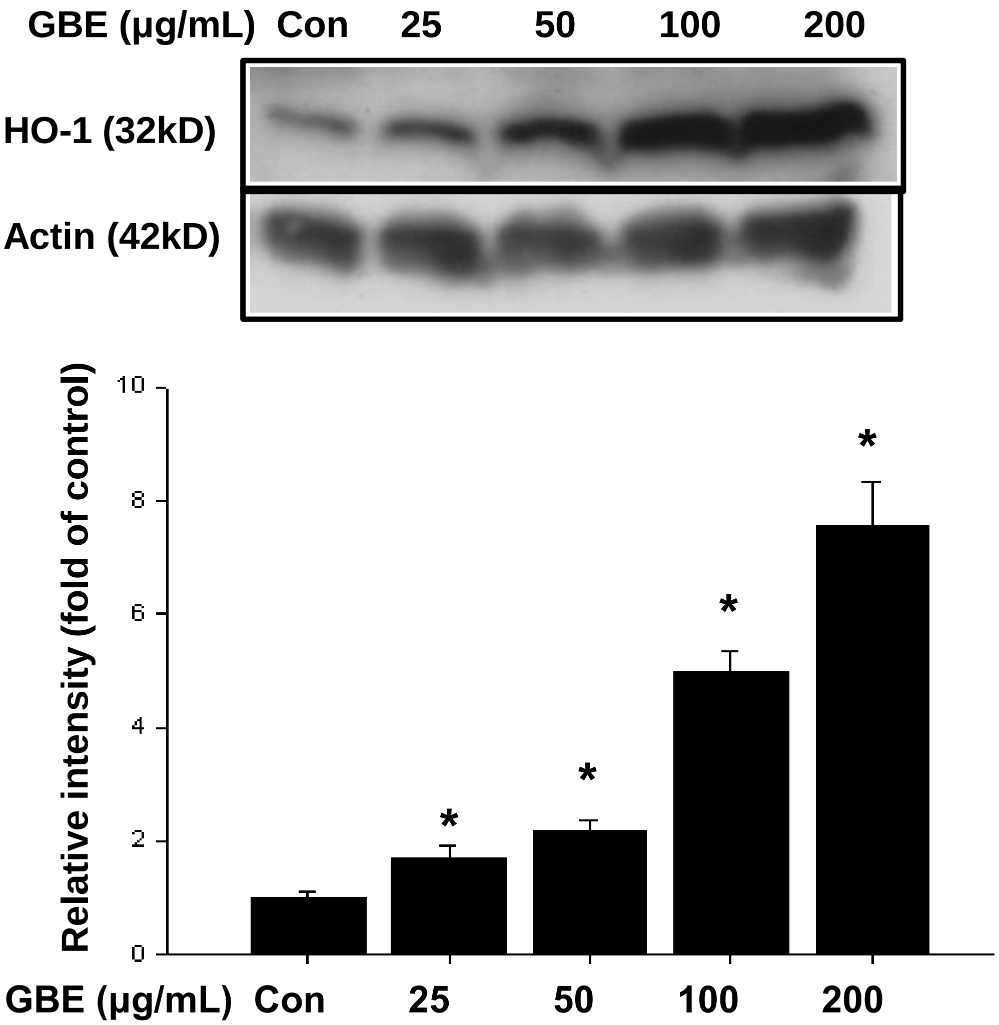

GBE (μg/mL) Con 25 50 100 200
HO-1 (32kD)
Actin (42kD)
Relative intensity (fold of control)
10
8
6
4
2
0
*
*
*
*
GBE (μg/mL) Con 25 50 100 200

Fig. 6D

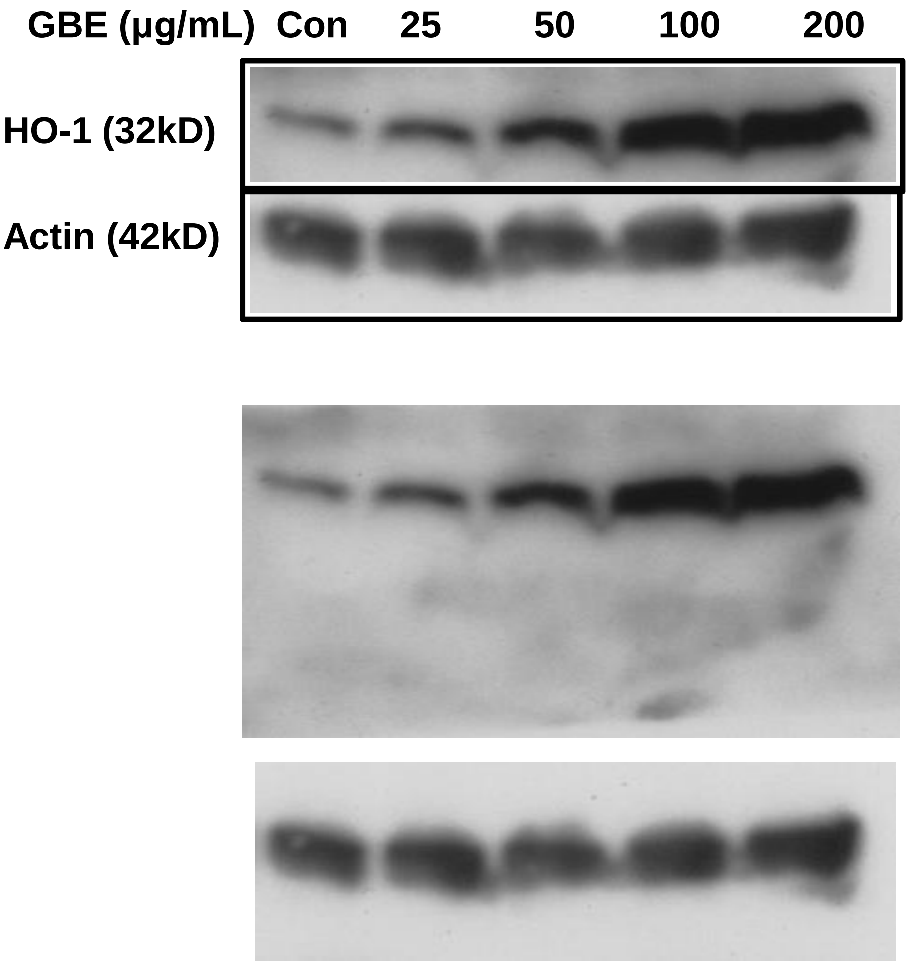

Fig. 7A

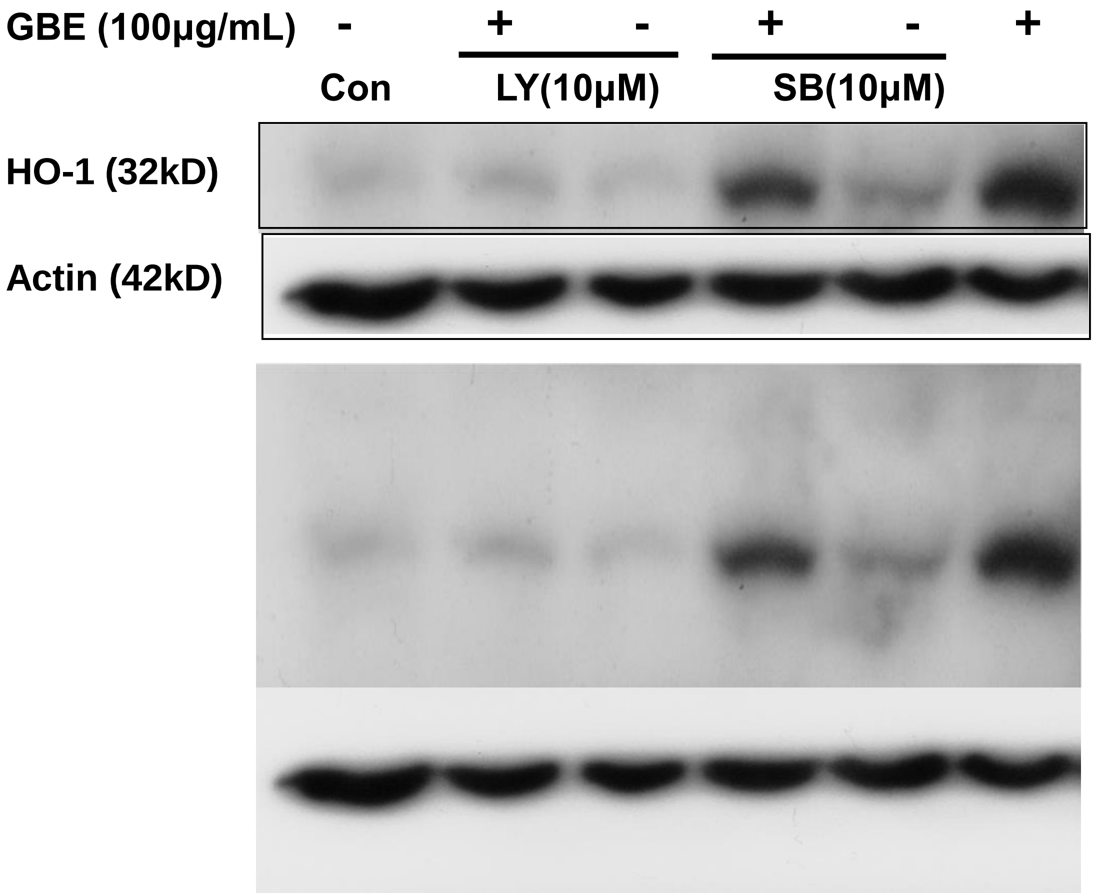

Fig. 7B

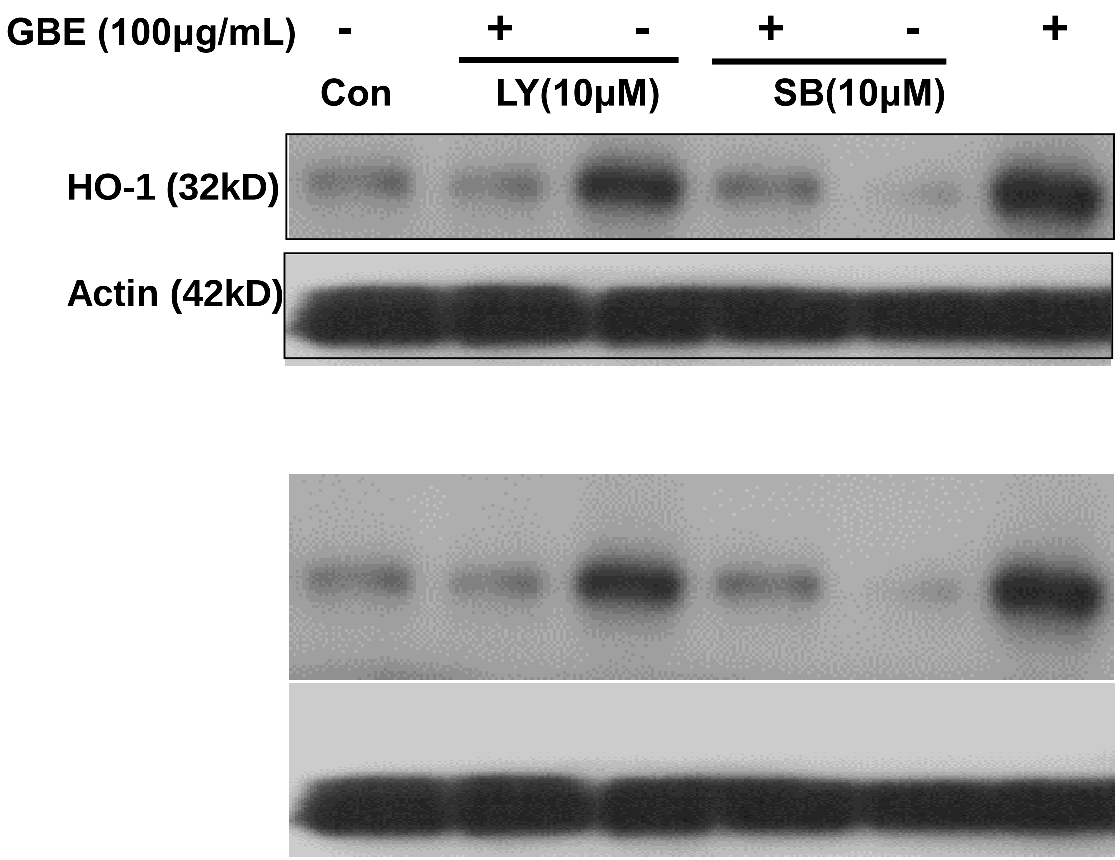

Supplementary Fig. 2

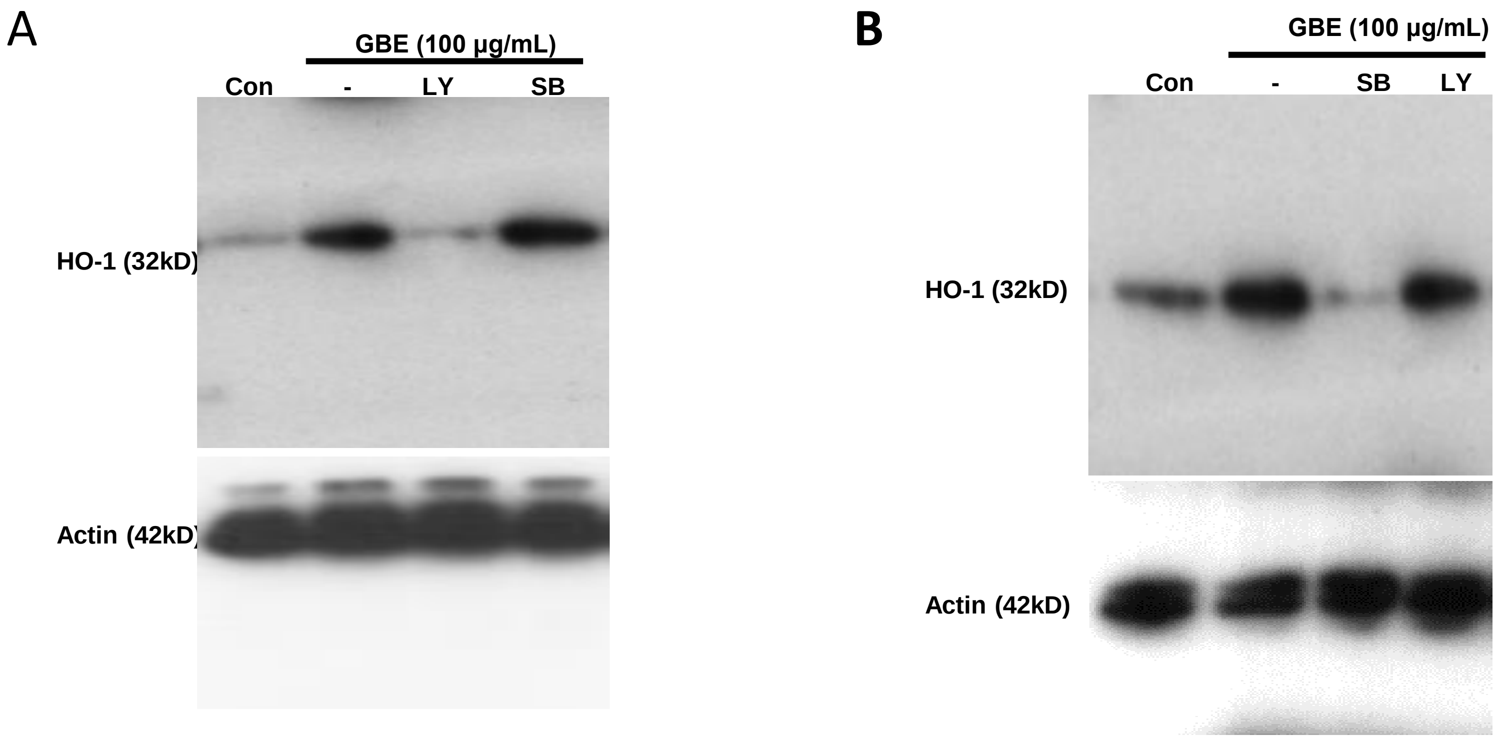

Supplement: Supplementary file 1 — Supplementary information [file 41598_2019_53818_MOESM1_ESM.pdf]
